# All-Atom Simulations Reveal the Intricacies of Signal Transduction upon Binding of the HLA-E Ligand to the Transmembrane Inhibitory CD94/NKG2A Receptor

**DOI:** 10.1021/acs.jcim.3c00249

**Published:** 2023-05-19

**Authors:** Martin Ljubič, Eva Prašnikar, Andrej Perdih, Jure Borišek

**Affiliations:** † National Institute of Chemistry, Hajdrihova 19, 1000 Ljubljana, Slovenia; ‡ Faculty of Pharmacy, University of Ljubljana, Aškerčeva 7, 1000 Ljubljana, Slovenia

## Abstract

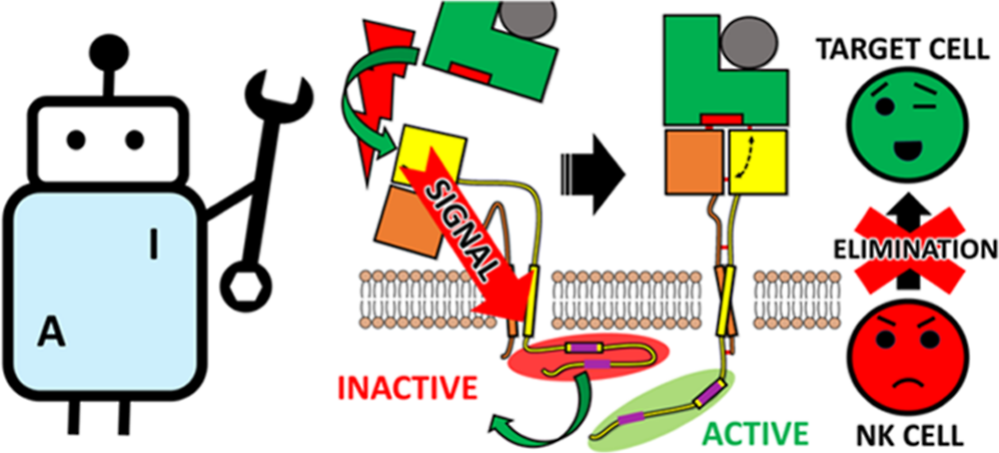

Natural killer (NK) cells play an important role in the innate immune response against tumors and various pathogens such as viruses and bacteria. Their function is controlled by a wide array of activating and inhibitory receptors, which are expressed on their cell surface. Among them is a dimeric NKG2A/CD94 inhibitory transmembrane (TM) receptor which specifically binds to the non-classical MHC I molecule HLA-E, which is often overexpressed on the surface of senescent and tumor cells. Using the Alphafold 2 artificial intelligence system, we constructed the missing segments of the NKG2A/CD94 receptor and generated its complete 3D structure comprising extracellular (EC), TM, and intracellular regions, which served as a starting point for the multi-microsecond all-atom molecular dynamics simulations of the receptor with and without the bound HLA-E ligand and its nonameric peptide. The simulated models revealed that an intricate interplay of events is taking place between the EC and TM regions ultimately affecting the intracellular immunoreceptor tyrosine-based inhibition motif (ITIM) regions that host the point at which the signal is transmitted further down the inhibitory signaling cascade. Signal transduction through the lipid bilayer was also coupled with the changes in the relative orientation of the NKG2A/CD94 TM helices in response to linker reorganization, mediated by fine-tuned interactions in the EC region of the receptor, taking place after HLA-E binding. This research provides atomistic details of the cells’ protection mechanism against NK cells and broadens the knowledge regarding the TM signaling of ITIM-bearing receptors.

## Introduction

Natural killer (NK) cells are large granular lymphocytes and, unlike the T and B cells, their activity does not require sensitization.^1^ They play an important role in the innate immune response against tumors and various pathogens such as viruses and bacteria, as well as in the regulation of the immune system through the production of cytokines such as interferon gamma (IFN-γ), an important activator of macrophages.^2^ NK cell functioning is controlled by a wide array of inhibitory [e.g., killer immunoglobulin-like receptors (KIR), Ig-like receptors (CD158), leukocyte inhibitory receptors (LIR1), and C-type lectin receptors (NKG2A-CD94)] and activating (e.g., NKG2D, NKG2C-CD94, NKp46, and 2B4) receptors, which are expressed on their cell surface.^3^

**Figure 1:**
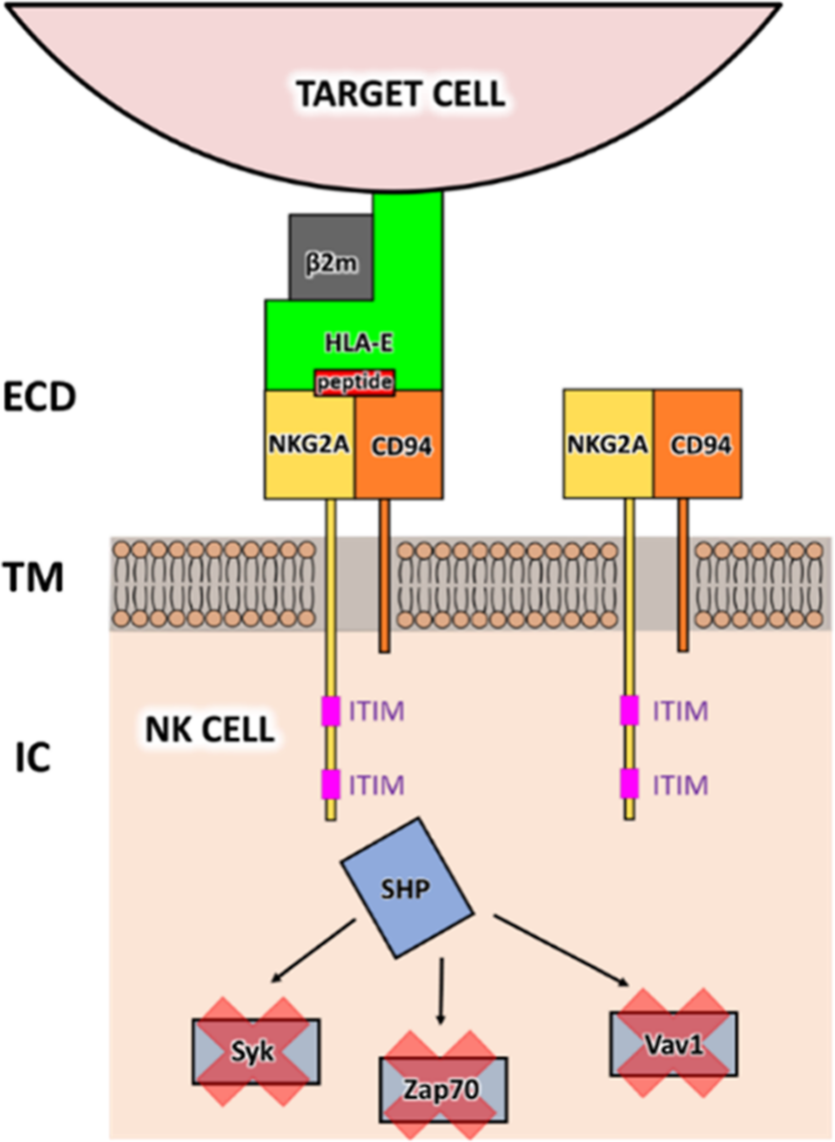
Schematic representation of the NKG2A/CD94 inhibitory receptor located on the surface NK cells. CD94 (orange) and NKG2A (yellow) are transmembrane proteins, the latter containing an intracellular domain with two ITIM regions. Target cells (e.g., cancer, senescent cells, and healthy cells) express HLA-E ligand (green) on their surface that binds specifically to the NKG2A/CD94 receptor together with its nonameric peptide (red), triggering receptor activation and downstream inhibitory cytotoxic effects through the SHP-induced dephosphorylation of signaling molecules (e.g., Syk, Zap70, and Vav1).

The inhibitory receptors on NK cells are responsible for preventing NK cell-mediated attacks on healthy cells, which express the MHC class I molecules for this purpose.^4^ Classical human leukocyte antigen (HLA) molecules generally exhibit a large degree of structural polymorphism, whereas the non-classical class Ib molecules such as HLA-E, HLA-F, and HLA-G have a more defined 3D structure. HLA-E is expressed on the cell surface as a trimeric complex consisting of the heavy and light chain of HLA-E, beta-2 microglobulin (β2m), and a specific nonameric peptide derived from the signal sequences of other HLA class I molecules (Figure 1).^5^

A variety of nonameric peptides allow receptor binding to HLA-E, but only a handful of them permit effective recognition, indicating that even a small change in their amino acid sequence can lead to a profound effect on their binding and molecular recognition.^6,7^ In the population, two alleles (HLA-E*01:01 and HLA-E*01:03) are dominantly expressed, differing only by one amino acid outside the peptide-binding cleft.^5^ In most human tissues, HLA-E is expressed at low levels, but we often find it overexpressed on the surface of different cancer as well as senescent cells, a type of cells that are in a state of stable cell cycle arrest resistant to growth-promoting stimuli, typically in response to DNA damage.^8^ As a result, the cytotoxic effector function of NK cells on these cells is severely impaired, and the cells can evade the immune system.

The HLA-E ligand binds specifically to the inhibitory CD94/NKG2 receptors with rapid association and dissociation rates and with higher binding affinities than when it interacts with the activating CD94/NKG2C receptor.^9^ It is likely that the signal transduction between HLA-E and the NKG2A/CD94 receptor primarily takes place via the α2 HLA-E domain, while the majority of energetically favorable interactions are established with the CD94 protein.^10^

Blocking the interaction between the inhibitory NKG2A/CD94 and HLA-E ligand has been shown to boost the immune response against senescent cells in vitro,^11^ revealing a possible mechanism how the immune system might regulate their presence in the organism. Furthermore, modulating this interaction might thus represent a promising new therapeutic strategy for tackling various age-related diseases such as dementia, hypertension, atherosclerosis, cancer, autoimmune, and other diseases by the enhanced immune clearance of senescent cells.^12^ Checkpoint inhibitors that target NKG2A, such as the antibody monalizumab, are already in clinical trials as potential new anticancer drugs.^13,14^

The dominant inhibition of cytotoxic action via the NK receptors such as NKG2A/CD94 heterodimer is carried out through a cytoplasmic immunoreceptor tyrosine-based inhibition motif (ITIM),^15^ which is constrained by the sequence S/I/V/LxYxxI/V/L, where x is any amino acid and Y is the tyrosine residue which can be phosphorylated by an appropriate protein kinase. The intracellular domain (ICD) of NKG2A contains two ITIM regions, separated by 26 residues (Figure 1). Studies on the platelet endothelial cell adhesion molecule-1 (PECAM-1) show that double ITIM phosphorylation is a sequential process, in which the Src family of protein kinases phosphorylate the C-terminal ITIM and allow for the subsequent N-terminal ITIM phosphorylation by other Src homology 2 (SH2) domain-containing non-receptor tyrosine kinases such as Lck and Fyn.^16^ In order for the maximal inhibitory function of NKG2A, activity of both ITIMs is required, although the membrane distal one was identified to be more crucial.^17,18^ The phosphorylation of ITIMs is followed by the recruitment of two Src homology region 2 domain-containing phosphatases (SHP), SHP-1 and SHP-2, which consist of two SH-2 domains. The removal of the autoinhibitory N-terminal SH2 domain activates the phosphatase catalytic domain. This is caused by binding to the phosphorylated ITIM tyrosine residue, which might require co-cross-linking the ITIM receptors with activating receptors.^18^ The result is the dephosphorylation of key molecules involved in cell activation such as Vav1, Zap70, and Syk.^19^

The CD94/NKG2 receptors span the membrane and are divided into three distinct regions: the extracellular domain (ECD), the transmembrane domain (TMD), and the ICD (Figures 1 and S1). The prevailing hypothesis is that ligand binding (e.g., HLA-E ligand) in the ECD induces stabilization of specific receptor conformations, but much is still unknown about the precise mechanisms that result in a signal being transmitted to the ICD.^20^ It is speculated that the key to signal propagation includes subtle changes in the TMD such as conformational changes or even oligomerization.^21^ This may be a consequence of the rearrangement of juxtamembrane domains in response to ligand binding. In the case of the NKG2A/CD94 receptor, the transduction of this signal is a necessary step in inhibiting the activity of NK cells and involves both NKG2A and CD94 components. Furthermore, signaling processes are not only dependent on the protein–protein interactions but can also be influenced by the lipid membrane by providing a surface with which proteins can interact. The composition of the lipid bilayer may therefore also exhibit a significant effect on the final outcome of the signaling cascade.^22^ Currently, various crystal structures of the NKG2A/CD94/HLA-E/β-2m complex are available,^23−25^ but to date, only the extracellular (EC) structure of the NKG2A/CD94 receptor has been resolved (Figure 1), and the structure of the complete receptor is yet to be determined. In addition, the molecular events associated with inhibitory signal transduction to the ITIM region after the CD94/NKG2 inhibitory receptor binds HLA-E are largely unexplored.

To further expand the knowledge concerning the NK inhibitory signal transduction via the NKG2A/CD94 receptor, we constructed the complete structure of the membrane-embedded NKG2A/CD94 receptor with and without the HLA-E/β2m ligand and nonameric peptide and used it in all-atom molecular dynamics (MD) simulations. Subsequently, we elucidated the main geometric and dynamic traits that could play an important role in the transduction of a signal through the lipid bilayer and provided a detailed atomistic outlook of the specific interactions and molecular events that guide the molecular recognition in NKG2A, as well as large-scale changes in receptor domain behavior. These results, furthermore, also shed light on the nature of immune receptor signaling in other ITIM-bearing receptors.

## Methods

### Structural Models of the Complete Immune Receptor and Its Complex with HLA-E

Structural models of the investigated immune complexes were built based on the crystal structure of the ECDs of the human CD94/NKG2A receptor in complex with the ECD of HLA-E [light (β-2m) and heavy chain] ligand and the leader nonameric peptide of the HLA class I histocompatibility antigen, alpha chain G, solved at a 3.4 Å resolution (PDB id: 3CDG).^23^ In addition to the EC structure, the immune complex consists of the TMDs and ICDs for which an experimentally solved structures are not available. For the prediction of the missing protein regions, we utilized ColabFold^26^ program in the form of the Google colab webpage, which employs AlphaFold2,^27^ a deep-learning algorithm and neural network-based model for the prediction of protein 3D structures based solely on the amino acid sequence. Sequence alignment and homology modeling for comparison with the AlphaFold2 structure were performed using SWISS-MODEL.^28^ Models were evaluated based on their predicted local distance difference test (pIDDT) scores,^29^ secondary structure prediction using PSIPRED4,^30^ and disorder prediction with DISOPRED.^31^

The available sequences of the transmembrane (TM) heterodimers NKG2A (residues 69–95) and CD94 (residues 9–33), which extend into the TM region, were used as ColabFold input along with the sequence of the cytoplasmic NKG2A region (residues 1–68), which contains the biologically relevant ITIM regions. After generating the structural models for the ECD, TMD, and cytoplasmic domains, we manually aligned them in ChimeraX^32^ and modeled the linker regions between domains de novo using MODELLER^33^ with the DOPE-HR loop modeling protocol to obtain the complete NKG2A/CD94 receptor structure.

Next, a 1-palmitoyl-2-oleoylphosphatidylcholine (POPC) membrane with 144 lipids per monolayer and a 65 Å^2^ area per lipid was generated using MemGen.^34^ The entire immune complex was then manually aligned to the membrane, and a 3 Å hole around the protein residue atoms was created to allow for adequate equilibration of the membrane lipids.^35^

Starting from the generated structure of the immune complex embedded into membrane, three models were constructed for molecular simulations. The first model (i) comprises HLA-E, β2m, NKG2A, and CD94 proteins and the G nonameric peptide (sequence: VMAPRTLFL), embedded in a POPC membrane, referred to as **COM**^**+**^ (Supporting Information files COM^+^.pdb, COM^+^.crd, and topology file COM^+^.top). The second model (ii) **COM**^**–**^ is identical to the first but lacks the G nonameric peptide. The third model (iii) **REC** is the apo form of the immune complex and contains only the NKG2A/CD94 receptor without HLA-E, β2m, and nonameric peptide.

### MD Simulations

Classical MDs simulations were performed using Amber 20 PMEMD software package.^36^ AMBER-ff19SB force field (FF) was used for proteins, and the Lipid17 FF was used for simulating the POPC membrane.^37,38^ Protonation states of ionizable residues were determined using the PDB2PQR web tool under a neutral pH condition of 7.^39^ Carboxylic amino acids were found in their common deprotonated states, whereas histidines were protonated at Nε, Nδ, or both positions. The system was solvated using Gromacs 2019^40^ in a layer of TIP3P water molecules according to the size of the lipid membrane, resulting in a box of 120.858 × 120.538 × 319.780 Å^3^. Water molecules inside the membrane were subsequently removed. Together with 14 Na^+^ counterions and water molecules, the entire system counted up to 415,920 atoms. Disulfide bonds were built using the *tleap* module of Ambertools 20,^36^ which was also utilized to prepare the topologies of the models.

The system was initially minimized in two steps using a steepest descent algorithm, followed by a conjugate gradient algorithm, with subsequent gradual heating to 300 K in a single step over 300 ps with positional restraints of 100 kcal/mol Å^2^ on the heavy atoms. Next, the restraints were removed, and five steps of 1 ns simulations of isothermal–isobaric ensemble (*NPT*) function ware performed, where pressure control (1 bar) was achieved using a Berendsen barostat^41^ to properly equilibrate the membrane lipids and periodic boundary conditions before the production step. The *skinnb* value during this step was increased to 5 to avoid any errors. Productive MD was then conducted in the *NPT* ensemble. To enhance the reliability of the performed simulations and better sample the conformational space, three replicas were simulated for each of the generated models.^42^ The duration of each simulation was between 1 and 2 μs for each replica, resulting in a total simulation time of ∼10 μs. During the MD simulations, temperature control (300 K) was performed using the Langevin thermostat^43^ with a collision frequency of 1 ps^–1^. The SHAKE algorithm^44^ was applied to constrain the bonds involved between hydrogen atoms and heavy atoms, and the particle mesh Ewald method^45^ with a cutoff of 10 Å was used to account for long-range electrostatic interactions. An integration time step of 2 fs was set during all MD runs.

### Analyses of Simulation Trajectories

Visual Molecular Dynamics (VMD),^46^ PyMol,^47^ and ChimeraX^32^ software packages were used for visualization and inspection of trajectories. MD trajectory analyses, including root-mean-square fluctuations (RMSF), and calculation of the cross-correlation matrices were performed with the *cpptraj*(48) module in Ambertools 20^36^ on the second half of the stripped trajectories without water and counterions. We excluded the first 500 ns of the trajectories to sample the generated conformational space as comprehensively as possible. Principal component analysis (PCA) was performed, and H-bonds were determined using the *cpptraj* module in Ambertools 20^36^ with cutoff of 3.0 Å and an angle cutoff of 135° as geometric considerations to account for a formed hydrogen bond. *Cpptraj* was used to analyze the degree to which the receptor head tilts in response to ligand binding. Two vectors, vertical and horizontal, were selected to represent both rotation angles. Angles between pairs of vectors were determined by *cpptraj* from the vector dot product with the unit cell vector. *Cpptraj* was also used to calculate the radial distribution functions (RDFs) for water molecules around selected protein atoms. The determination of secondary structure changes throughout the simulation was performed using the DSSP algorithm in *cpptraj*(49)

### Cross-Correlation Matrices and Correlation Scores

The cross-correlation matrices, based on the Pearson’s correlation coefficients (CCij), quantify correlated and anti-correlated motions between the pair residues along the MD trajectory. CCij values can range from −1, indicating completely anti-correlated motion between two residues, to +1, indicating correlated motion; meanwhile, 0 indicates no correlation. The Pearson correlation coefficient (*C*_*ij*_) is computed on the Cα atom pairs, *i* and *j* via eq 1.
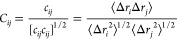
1

In the equation, *c*_*ij*_ is defined as *c*_*ij*_ = ⟨Δ*r*_*i*_Δ*r*_*j*_⟩, and Δ*r*_*i*_ represents a displacement vector of atom *i* and Δ*r*_*j*_ of atom *j* with the brackets signifying an ensemble average.

First, the covariance matrices were built from the atom position vectors using Cα atoms of the protein backbone. To capture only the internal dynamics of the complex, RMS-fit to a reference structure (an averaged structure from the MD run) was performed, removing the rotational and translational motions as previously described.^50−52^ Next, the cross-correlation matrices (or normalized covariance matrices) were calculated from the covariance matrices in the *cpptraj* module of Amber tools 20.^36^ To illustrate simplified relationships between individual proteins and domains of the immune complex, the correlations for each protein/domain pair were assessed by summing the correlation scores (CSs) between each protein/domain and all others. Next, a correlation density for each area was obtained by summing the CSs of the protein/domain pair, which was then divided by the product of the number of residues belonging to that pair of proteins/domains, thus obtaining a simplified variant of the CCij matrices. We also calculated differential correlation matrices by subtracting values at the same positions between matrices of two models to help illustrate differences in correlations.

### Principal Component Analysis

PCA^53^ was performed in cpptraj to extract the essential dynamics of the proteins, starting from the mass-weighted covariance matrix of the Cα and P atoms, respectively. The covariance matrices were built from the atoms position vectors upon an RMS-fit to the reference starting configuration of the MD production run in order to remove the rotational and translational motions. The eigenvectors with the largest eigenvalues correspond to the direction of the most relevant motions sampled during the simulation, which is also referred to as principal components (PCs). By projecting the displacement vectors of each atom along the trajectory onto the eigenvectors, it is possible to reduce the dimensionality and the noise inherent in a trajectory and thus to obtain only the most relevant motions. The cumulative variance accounted by the PCs was calculated for all three models with Gromacs 19.^40^ The Normal Mode Wizard plugin^54^ available within the VMD program^46^ was used to visualize the essential dynamics along the principal eigenvectors and to draw the arrows highlighting their direction.

### Binding Free-Energy Calculations

Binding free energies between the complexes HLA-E/β2m/peptide/NKG2A/CD94 and the corresponding nonameric peptide, and between NKG2A/CD94 (receptor) and HLA-E/β2m/peptide (ligand), as well as between NKG2A and CD94 components of the receptor, were calculated using the Molecular Mechanics/Generalized Born Surface Area (MM-GBSA) method^55^ and Amber20 code.^36^ The value of the igb flag was set to 5, and a salt concentration of 0.1 M was used, as used in many other MM/GBSA calculations.^56^ MM-GBSA calculations were performed for 100 equally distant frames from each MD trajectory in the production simulation time interval between 500 and 2000 ns. The energy values were all averaged from the three replicas of the systems. The conformational entropic contribution of free energy was not included in the calculations since it was previously suggested that this term does not improve the quality of the results when using MM-GBSA.^57^

## Results

### Complete Immune NKG2A/CD94 Receptor Model and Its Complex with HLA-E

The complete immune inhibitory NKG2A/CD94 receptor model comprises the crystallographically determined EC section,^23^ along with the ICDs and TMDs, for which experimentally solved structures are not available. We hence generated suitable 3D models of the NKG2A and CD94 TM helices (residues 69–95 and residues 9–33, respectively) by a ColabFold program with a predicted IDDT overall score of over 80% (Figure S2) with this value dropped to 70% at both ends of the helices, indicating a degree of uncertainty of the side chain positioning and helix length at these locations. Upon aligning the homology-modeled helices and helices generated by Alphafold2, the observed differences between the models were not significant and were apparent only at both edges of the helices, terminal two residues (Figure S3). The conformation of the backbone was identical, and the positioning of side chains was comparable between both models. Furthermore, the Alphafold-generated dimer contained an orientation of cysteine residues, which allowed the formation of a disulfide bond between cysteines Cys80^NKG2A^–Cys20^CD94^. While the prediction of multi-chain complexes using Alphafold 2 is challenging and often does not result in models with high accuracy,^58,59^ the resulting TM complex was stable in the proceeding simulations and was able to reflect changes in the receptor behavior in the complete receptor structure. The presence of this disulfide bond may somewhat restrict the dynamics of the receptor TM region. In spite of this conformational restriction, the simulations were still able to display changes in helix positioning as a result of HLA-E binding.

Next, the remaining ICD of the NKG2A protein (residues 1–68) was modeled using the same approach as utilized for the TM domain. Over 800 homologous sequences of this domain were found during the model generation. Secondary structure prediction and disorder prediction indicated a largely unstructured domain (Figure S4). However, Alphafold2 generated a helical structure at the position of the C-terminal ITIM. Interestingly, this prediction was corroborated by PSIPRED4, which predicted an α-helix in this region as well. DISORDPRED results also suggest that the likelihood of disordered structure in this region was smaller. The pIDDT score for the highest scoring model was 50% for the unstructured regions and jumped to 80–90% at the location of the α-helix located at position Tyr40 (Figure S5).

### Global Changes in Protein Domains Dynamics and Possible Signal Transduction

**Figure 2:**
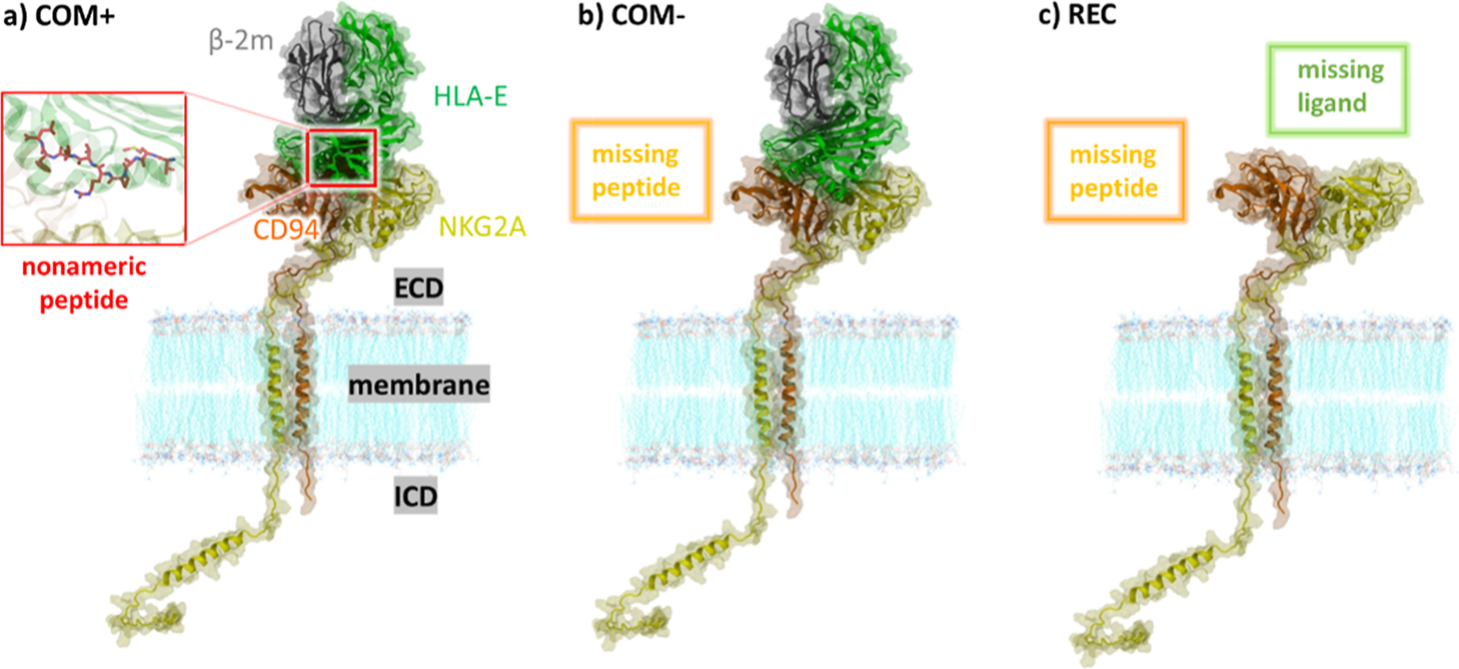
Complete inhibitory immune receptor models and its complex with HLA-E, built from the solved extracellular structure (PDB id: 3CDG) and Alphafold-generated ICDs and TMDs used in molecular simulations. They consist of NKG2A (yellow), CD94 (orange), HLA-E (green), β-2m (gray), and nonameric peptide (red). Left: complete immune complex model (**COM**^**+**^); middle: complete immune complex without a nonameric peptide (**COM**^**–**^); and right: transmembrane receptor NKG2A/CD94 without ligand and peptide (**REC**). Shown at the bottom for all models is the ICD of NKG2A with N-terminal and C-terminal ITIM regions colored in magenta.

To investigate the behavior of the constructed complete immune NKG2A/CD94 receptor in the absence of its ligand and in the case when the HLA-E ligand is bound to the NKG2A/CD94, we subsequently conducted several 1–2 μs long MD simulations (Figure 2 and Movie S1). In cases where this was possible, we have presented the results as an average of three independent simulations/replicas of each model. Otherwise, the figures are based on the results of the initial run/replica (i.e., time-dependent plots, PC1 projections, and angular analysis).

We assume that upon binding (e.g., the HLA-E ligand), the ligand recognition enables further conformational changes, leading to the alterations in the key intracellular ITIM regions, which subsequently allow them to be phosphorylated and act as binding sites for the SHP phosphatase.

**Figure 3:**
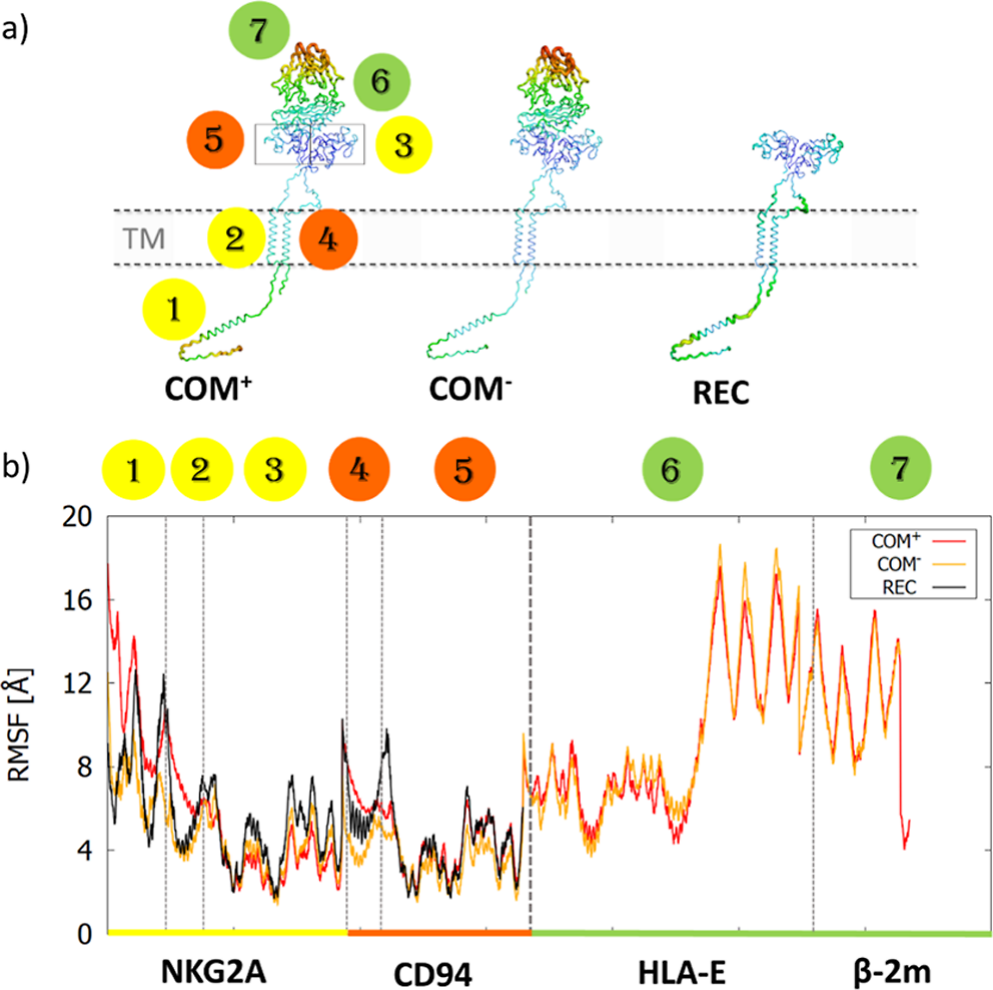
Flexibility of the protein domains depicted as the (a) B-factor representations and (b) RMSF plot for all three simulated models. Only the receptor structure was used in the alignment process. Domains are marked with numbers and separated with dashed lines in the RMSF graph: (1) IC NKG2A, (2) TM NKG2A, (3) EC NKG2A, (4) TM CD94, (5) EC CD94, (6) HLA-E, and (7) β-2m.

Observed average root-mean-square deviation (RMSD) values for **COM**^**+**^, **COM**^**–**^, and **REC** models were 20 ± 5, 23 ± 3, and 27 ± 2.5 Å, respectively, which stabilized after 200 ns, however still fluctuated in the equilibrated part of the trajectories (Figure S6). Comparing only the EC receptor regions of the models, the RMSD values in all models are close to 3 Å and remain reasonably constant, indicating that no large-scale changes within the receptor head occurred during the simulation (Figure S7). Next, RMSF analysis was performed to further evaluate the effects of ligand binding on the flexibility of the receptor. This showed that in the absence of bound HLA-E ligand, the receptor exhibited higher flexibility in the EC region of NKG2A as well as in both linker regions of NKG2A and CD94 protein (Figure 3).

On the other hand, the opposite outcome (lower flexibility) was noticed for the TM and IC domains, except for the first five residues of CD94, which are not embedded in the membrane. The most notable difference in the IC domain was detected in the first 15 residues, where the flexibility in **COM**^**+**^ outweighed the **REC** system by a factor of 2. Interestingly, while the RMSF values of the **COM**^**–**^ system were similar to **COM**^**+**^ in other regions, **COM**^**–**^ remained less flexible in the IC region of NKG2A, the part of the receptor which was suspected to importantly regulate in the signal transduction.^10^ Slight differences between the **COM**^**+**^ and **COM**^**–**^ were also observed in the flexibility of the HLA-E ligand. In the absence of the nonameric peptide, parts of HLA-E were more flexible in the **COM**^**–**^ model, which may indicate a stabilizing effect of the peptide on the complex, as reported previously.^7^

**Figure 4:**
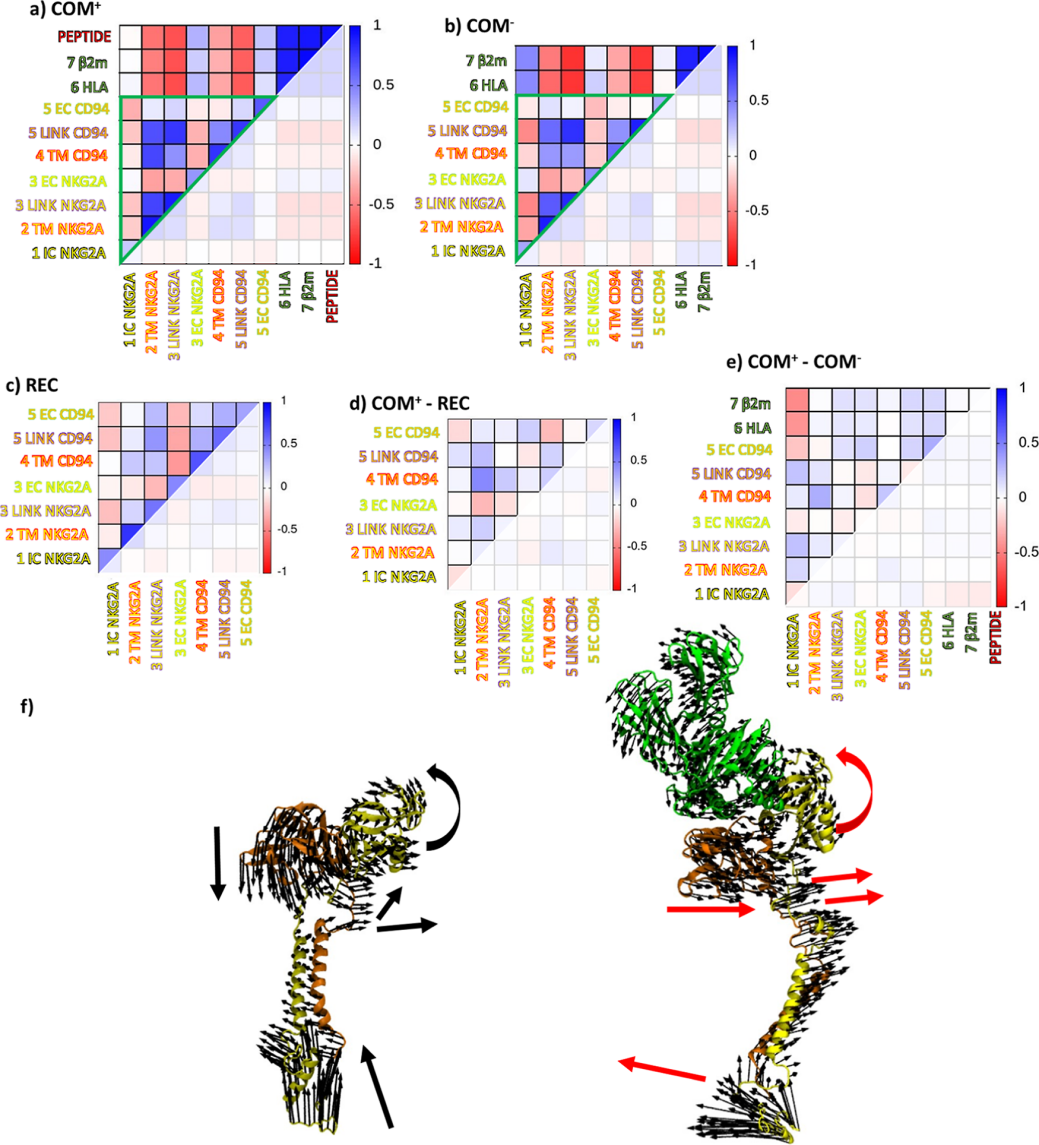
Global dynamical analysis of the immune complexes. Simplified cross-correlation matrices for simulated models (a) **COM**^**+**^, (b) **COM**^**–**^, (c) **REC**, and (d) differential matrix, calculated as the difference between models **COM**^**+**^ and **REC**. (e) Differential matrix, calculated as the difference between models **COM**^**+**^ and **COM**^**–**^. (f) Projections of the first principal mode PC1 onto the 3D structures of **COM**^**+**^ (right) and **REC** (left). Large arrows discern global motions of the protein.

To evaluate the correlations between the motions of protein domains, dynamical cross-correlation matrices were constructed from averaged data from multiple replicas. Correlation matrices of **COM**^**+**^ and **COM**^**–**^ contained similar patterns with the most pronounced linker and TM regions, which were all strongly correlated. Instead, EC regions of CD94 and NKG2A were less correlated with the remainder of the protein structure, with the EC NKG2A even displaying anticorrelations with the linker and TM domains. The HLA-E ligand was strongly anticorrelated with these regions as well but was slightly correlated with the receptor head. In the **REC** correlation matrix, NKG2A and CD94 ECDs were anticorrelated. The correlations between linker and TM domains are also less apparent. According to the differential matrix, ligand binding induces stronger correlations between linker and TM regions of CD94 and NKG2A. A positive correlation between two parts of the receptor head, together with a positive correlation with HLA-E, shows that the receptor ECD becomes stabilized and allows for further communication between NKG2A and CD94. The anticorrelation of EC and TM NKG2A might indicate a change in TM behavior as a result of this communication. The differential matrix between **COM**^**+**^ and **COM**^**–**^ contained less pronounced differences than between **COM**^**+**^ and **REC** (Figures 4a–e and S8).

In order to isolate the main structural elements of these large-scale collective motions, we performed the PCA of the obtained MD trajectories (Figure S9). From the PCA, we extracted the essential dynamics of the systems,^60^ where the motion of the system was projected onto the first PC, and no significant differences were observed between the **COM**^**+**^ and **COM**^**–**^ models.

On the other hand, several regions of the NKG2A/CD94 receptor were observed to behave differently without the presence of the HLA-E ligand. The EC regions of CD94 protein eigenvectors point in a vertical direction in the unbound state but are more horizontal in **COM**^**+**^ and **COM**^**–**^. This implies that CD94 and NKG2A move in a lockstep motion, induced by interactions with the HLA-E ligand. Additionally, the eigenvectors of the CD94 regions which link the ordered EC and TMDs (“linkers”) point in the same direction in the **COM** models as opposed to **REC**, where this is not as apparent (Figure 4f). The observed effects were also noted in the replicas of the systems.

One indicator that the key ITIM regions in NKG2A may position differently as a result of the HLA-E binding was detected in the IC region of the NKG2A protein, in which the eigenvectors pointed in a horizontal direction in **COM**^**+**^ and vertical in **REC**, located in the same region where the differences in RMSF were most apparent. These results provided first indications that the binding of HLA-E to the complete NKG2A/CD94 receptor leads to substantial domain dynamics that could be linked to the signal transduction.

Altogether, the observed global changes indicate differences between the **COM** and **REC** models. In the following Results subsections, we individually examined the IC, TM, and EC regions of the receptor in search for potential key signaling events that would ultimately allow the construction of a model of signal transduction, which will be presented in the Discussion section.

### HLA-E Binding Induces Changes in the Positioning of the Intracellular NKG2A ITIM Region

**Figure 5:**
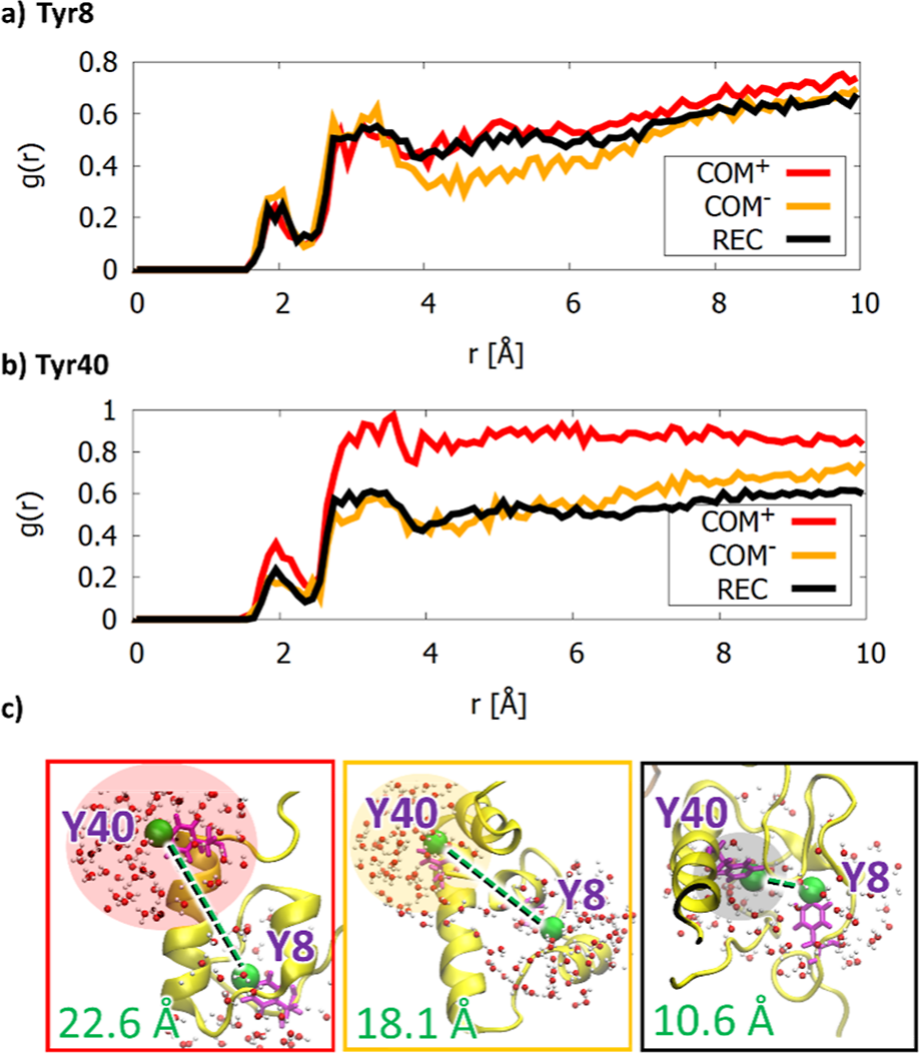
RDF of water molecules around the ITIM tyrosine oxygen (OH) atoms of (a) Tyr8 and (b) Tyr40. Images under (c) showcase the distribution of water molecules around both ITIM regions and the average distances between tyrosine OH atoms for all the subjected models. While no significant difference is observed for Tyr8, the RDF function for Tyr40 of **COM**^**+**^ stands out significantly. The key difference is visualized in the red (exposed Tyr40 of **COM**^**+**^), orange (semi-exposed Tyr40 of **COM**^**–**^), and black boxes (sheltered Tyr40 of **REC**).

Coupled with global changes in the immune complex protein structure and dynamics, we also observed further interesting changes in the IC region of the NKG2A protein. Distance analysis between the ITIM Tyr8 and Tyr40 side-chain oxygen atoms revealed that the average distance calculated from three replicas between their oxygen atoms of the side-chain OH group is 22.6, 18.2, and 10.6 Å for **COM**^**+**^, **COM**^**–**^, and **REC**, respectively, pointing to a noticeably higher distances between these tyrosines in the models with bound HLA-E ligand (Figure 5).

In addition, after the initial equilibration phase, the distance between the Tyr8 and Tyr40 residues remains predominantly constant throughout the simulation (Figure S10). This suggests that the intracellular region of the NKG2A can form more stable conformations that retain such geometric parameters, although this region of the receptor is much more mobile than other protein domains. Visual inspection further showed that the relative orientation of the two ITIMs was different depending on the model, which was most evident when comparing the orientations of the Tyr8 and Tyr40 OH atoms (Figure 5).

Upon the examination of the secondary structure changes in the NKG2A IC and TM regions taking place during the simulations with the DSSP algorithm, we observed for the **COM**^**+**^ and **REC** that parts of the IC helix disappeared (Figure S11). In **REC**, this structural change occurred immediately, even before the main production part of the simulation and after that, it remained stable, and its size remained halved until the end. This helix always contained the C-proximal ITIM region, and around the N-terminal ITIM, a small helical structure was also observed to form. Similar to **REC**, the helix in **COM**^**+**^ shrank during the simulation, but this happened at a later stage of the production run of the simulation. The helix also broke apart at location of the C-terminal ITIM. Changes in the secondary structure have been observed experimentally in other immune receptors, such as the formation of dynamical alpha helices upon interactions with a lipid bilayer, which was hypothesized as one possible mechanism of signal transduction.^61^

RDFs of the water molecules surrounding the Tyr40 tyrosine oxygen atoms show a stark contrast between **COM**^**+**^ and the other two models (Figure 5b). Below 3 Å, the first peak indicates a slightly higher exposure to water molecules of Tyr40 in **COM**^**+**^ and the difference balloons to 0.3–0.4 above 3 Å. The same trend was not observed for the N-terminal ITIM Tyr8 oxygen atoms, whose water RDF functions are comparable between all three simulated models in the entire graph region (Figure 5a).

Visual inspection revealed that the Tyr40 residue is shielded from waters by the rest of the intracellular region in **REC**, whereas in **COM**^**+**^, in which the HLA-E is bound to the NKG2A/CD94 receptor, it remains exposed to the surrounding water core. Another noticeable difference between **COM**^**+**^ and **REC** is that the ITIM regions seem to be located further away from the membrane in the case of **COM**^**+**^ with an exception of one replica, in which the IC helix interacted with the membrane more frequently. Distance analysis between centers of mass of residues 1–45 and the TM helices (residues 73–95 of NKG2A and 1–33 of CD94) showed that the **COM**^**+**^ ITIMs were farther away from the TM regions than that in **REC** (Figure S12). Overall, it was observed that the IC region of NKG2A fluctuated more after HLA-E binding, both in the terms of proximity to the TM regions and in terms of the number of contacts with the membrane.

To evaluate membrane interactions between the ITIM-containing IC region (residues 1–45) of NKG2A and the membrane, we calculated the number of contact pairs during each simulation frame (Figure S13). In **REC**, a number of contacts remained consistent throughout the entire trajectory. In **COM**^**+**^, the fluctuations were larger, displaying both zero contacts and significant peaks with transient strong contacts. This may be another indicator that the ITIM portions of the receptor are more flexible as a result of ligand binding.

### Transmembrane Helices of NKG2A and CD94 Are Poised to Transmit a Signal across the Lipid Bilayer

**Figure 6:**
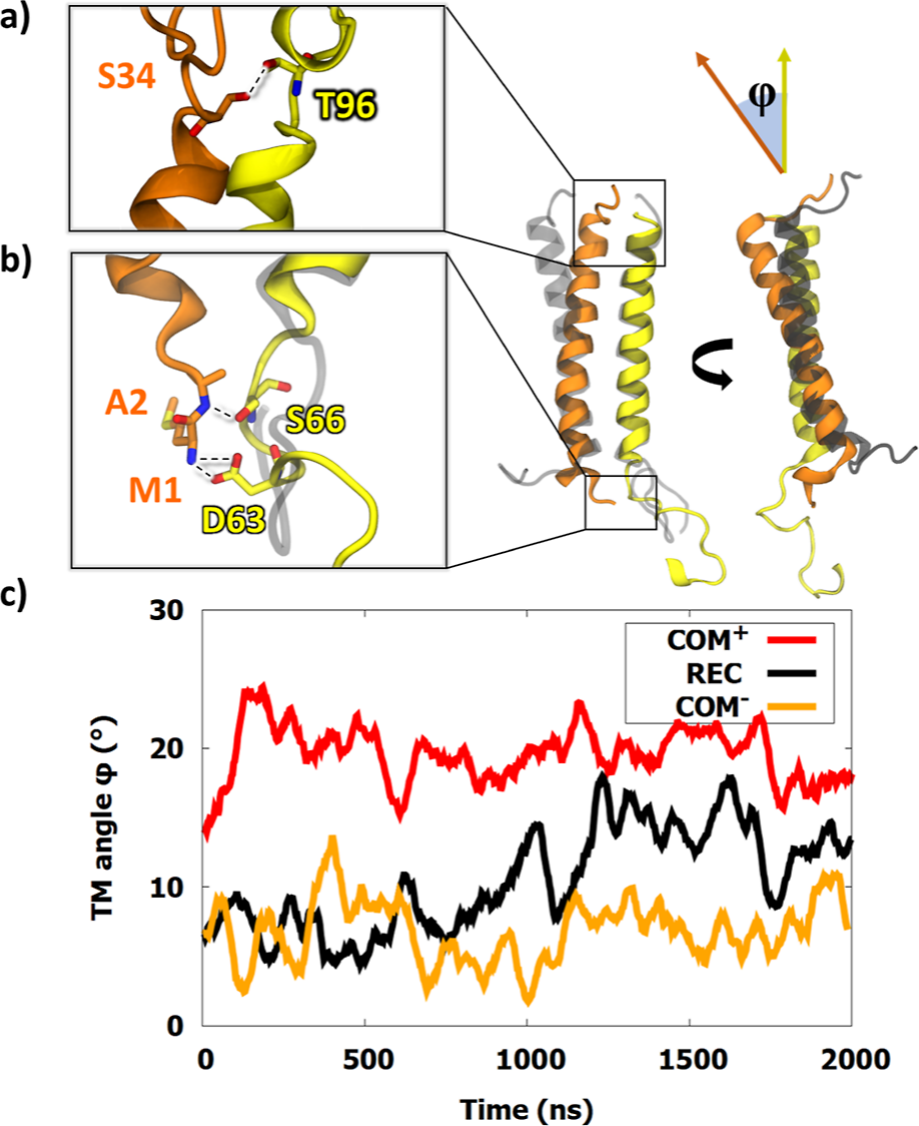
Changes in the TMD orientations after HLA-E ligand binding. TM helices of NKG2A (yellow) and CD94 (orange) of **COM**^**+**^ are colored. Gray helices represent **REC**, aligned to the **COM**^**+**^ dimer structure. The right image is rotated 90° around the vertical axis. Membrane lipids are not shown. (a) Hydrogen bonds Thr96^NKG2A^-Ser34^CD94^ and (b) hydrogen bonds Ser66^NKG2A^-Ala2^CD94^ and Asp63^NKG2A^-Met1^CD94^. (c) Comparison of the angle between helices as a function of simulation time, calculated from representative vectors, schematized in the figure as φ.

In most TM receptors such as GPCRs, the TMDs act as mediators in the signal transduction after ligand binding.^62^ Consequently, we also investigated how the NKG2A/CD94 helix dimer changes upon the HLA-E binding and established that the angle between the helices, calculated from the Cα atoms on both ends, significantly differed between the **COM**^**+**^ and **REC** systems (Figure 6). In the latter, this angle is smaller by at least 10° on average. The helices also adopt a different orientation: a crossed shape in **COM**^**+**^ and a Y shape in **REC**. In **COM**^**–**^, the TM angle is the smallest, but the helices still adopt a crossed shape, more similar to **COM**^**+**^. Although the CD94 molecule is similarly bent near the intracellular part of the membrane in both **COM** and **REC** models, its five-residue intracellular tail protrudes from the bottom of the membrane at a different angle in **REC**. As we will see in our constructed model of signal transduction, we could link this behavior of the TM helices to the observed changes in the IC region.

To follow-up on this observation, we calculated the distance between the centers of mass of residues 1–3 of CD94 and 63–53 of NKG2A protein and found a moderate difference in distance during the first part of the simulation, with **COM**^**+**^ even dropping under 5 Å while **REC** stays at a consistent 15 Å (Figure S14). However, after 1 μs of the simulation, a significant change occurred in **REC**, in which this monitored distance spiked to 30 Å. During the entire simulation, the distance was smaller in **COM**^**+**^ and **COM**^**–**^, which might point toward interactions between the five-residue intracellular part of CD94 and the membrane-proximal 10–15 residue region of the NKG2A ICD. This observed structural feature dependent on the HLA-E ligand binding could be associated with the necessary molecular machinery of signal transduction taking place via the NKG2A/CD94 receptor. Analysis of the contacts between these two TM regions of CD94 and NKG2A identified two possible hydrogen bonds formed between the Met1^CD94^-Asp63^NKG2A^ and Ala2^CD94^-Ser66^NKG2A^ (Figure 6).

These two bonds occurred transiently in **COM**^**+**^ but not in **REC**. Hydrogen bond analysis in the linker regions of NKG2A and CD94 further identified a hydrogen bond between Ser34^CD94^-Thr96^NKG2A^ at the top of the TM helices in **COM**^**+**^ (Figure S15). The initial average distance between residues was 9 and 13.5 Å for **COM**^**+**^ and **REC**, respectively. This interaction occurred only after 1.2 μs in **COM**^**+**^, yet was present in all observed frames after its formation.

Analysis of the total number of atomic contacts between the two linker regions revealed that the number of contacts was higher in **COM**^**+**^ than **REC** (Figure S16). This indicates closer proximity and more interactions between the juxtamembrane parts of NKG2A and CD94. Additionally, **COM**^+^ was found to form fewer contacts between the linker regions and membrane lipids, indicating that these regions are more exposed to water molecules and less proximal to the membrane in the **COM** models than that in **REC** (Figure S17).

### Ligand Binding Restricts the Conformational Dynamics of the NKG2A/CD94 ECD, and Fine-Tuned Interactions Mediate Their Molecular Recognition

**Figure 7:**
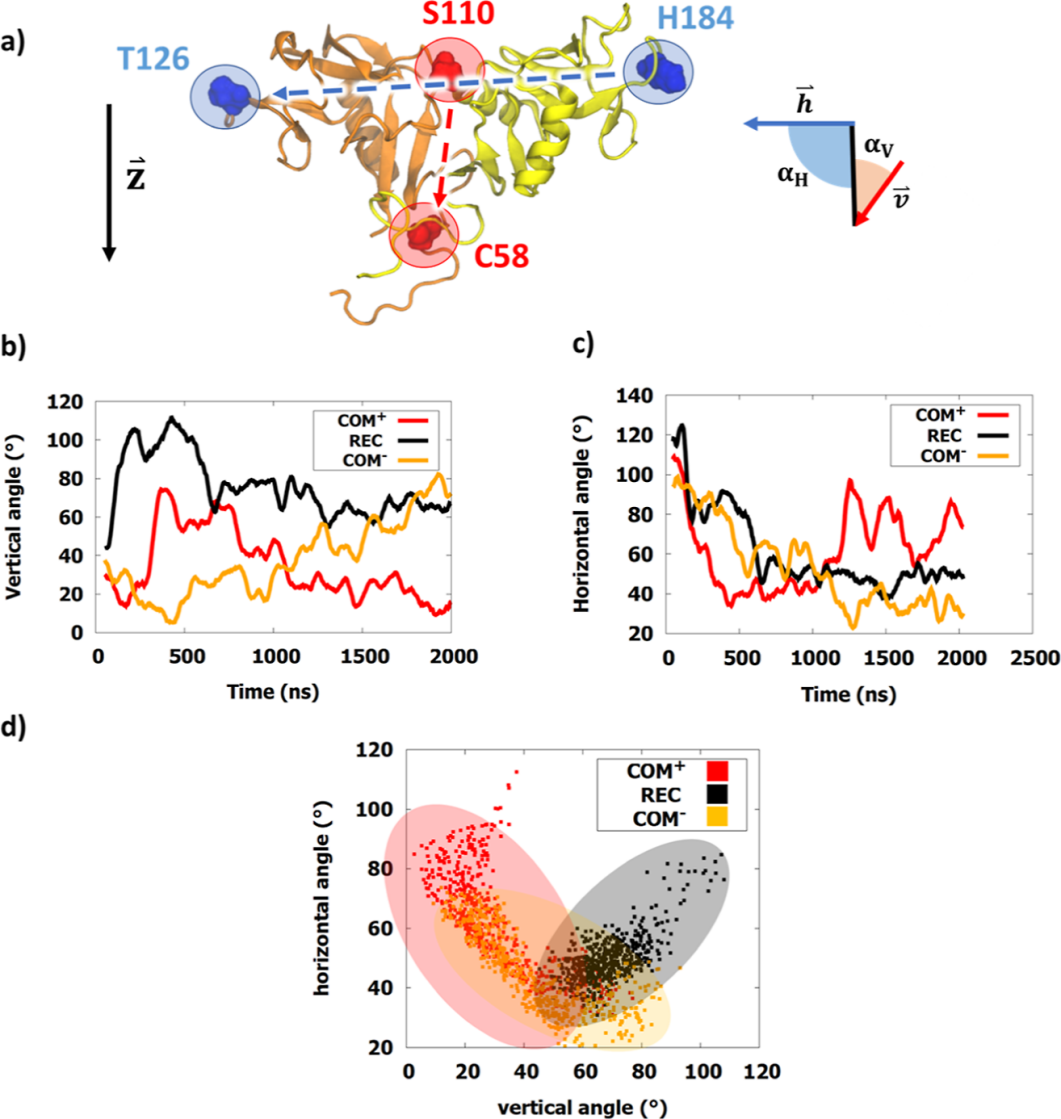
Bending angle analysis of the extracellular NKG2A/CD94 receptor domain. Angles were calculated with respect to the vertical unit cell vector . (a) Visual representation of the selected vectors. The vertical vector is shown in red (), while the horizontal vector is colored in blue (). The schematic on the right shows the horizontal (α_H_) and vertical (α_V_) angles. (b) Vertical angle, using vector between the centers of mass of Cys58-Ser110. (c) Horizontal angle, using vector His184-Thr126. (d) 2D angle plot, showing the conformational space of the receptor head. The **COM**^**+**^ space is highlighted in red, while **REC** is highlighted in gray.

The ECD of the NKG2A/CD94 receptor plays a crucial role in proper molecular recognition of the HLA-E ligand with bound nonameric peptide. The changes in this region following ligand binding represent the first step in signal transduction and are highlighted along with other key events in the Discussion section. To better understand its global dynamics with respect to the lipid bilayer, we monitored it via two bending angles, which together map the positioning of the receptor ECD relative to the membrane (Figure 7).

In the analysis of these angles, we noticed significant differences in the range of horizontal and vertical angles between **COM**^**+**^ and **REC** (Figure 7). The **REC** system initially displayed a horizontal angle much closer to the starting structure (Figure 2C), in which the receptor ECD was perpendicular to the lipid bilayer at 90°. Eventually, it settled at a 70° vertical angle, indicating a large tilt of the receptor head. The presence of bound HLA-E in **COM**^**+**^ caused a smaller degree of tilt along both angles. The horizontal angle varied from 40 to 100°, but no long-term protein–lipid interactions were observed. While comparing the vertical angles, the opposite effect was observed. The **COM**^**+**^ receptor head domain settled at under 40° and remained consistent during the second half of the trajectory, indicating a more upright position closer to 0°. The **COM**^**–**^ model behaved similar to **REC** toward the end but was able to occupy smaller vertical angles throughout the simulation like **COM**^**+**^.

Much more extensive changes observed in **REC** past the equilibration phase indicate that HLA-E binding to NKG2A/CD94 alters the receptor positioning in the ECD (Movie S1). By plotting pairs of vector angles on a 2D diagram, we compared the clustering of points between **COM**^**+**^ and **REC** (Figure 7). Points belonging to the **REC** system without the bound HLA-E spanned a smaller interval of horizontal angles. Although the two conformational clusters intersected, substantially lower vertical angles were accessible to **COM**^**+**^. Upon visual inspection of the obtained trajectories, this is likely due to increased interactions of the EC portions NKG2A and CD94 with the lipid polar groups in the **REC** models, which stabilize a significant tilt along one axis. For the HLA-E to successfully bind to NKG2A/CD94, these contacts must be abolished. **COM**^**–**^ contained sets of angles that intersected with both models, but displayed lower vertical angles than **REC**, indicating the same vertical lowering effect as seen in **COM**^**+**^.

We also investigated the number of contacts between the ECD of NKG2A/CD94 and membrane lipids and found that **COM**^**+**^ formed fewer contacts with the membrane lipids than **REC** (Figure S18), indicating that membrane contacts may play a role in signal transduction from the ECD to the TM regions.

The studies of the molecular recognition between the HLA-E and the EC portion of the NKG2A/CD94 receptor indicated that the hydrogen bonds formed between the Lys_135_^NKG2A^–Asp_106_^CD94^ and Lys_135_^NKG2A^–Ser_109_^CD94^ were more pronounced in the presence of the HLA-E ligand.^10^ Having the complete NKG2A/CD94 receptor at hand, we analyzed the frequency of these interactions for each of the three models and found that the percentage of frames in which the Lys_135_^NKG2A^–Ser_109_^CD94^ distance was below 4 Å for **COM**^**+**^, **COM**^**–**^, and **REC** was 57.7, 54.5, and 14.2%, respectively. For Lys_135_^NKG2A^–Asp_106_^CD94^, the percentages were 38.5, 49.5, and 15%. This bond thus formed even more frequently in **COM**^**–**^ than **COM**^**+**^, although this is likely that longer sampling would lead to the abolition of this anomaly. Importantly, in the absence of HLA-E binding, the two hydrogen bonds were far more infrequent.

**Figure 8:**
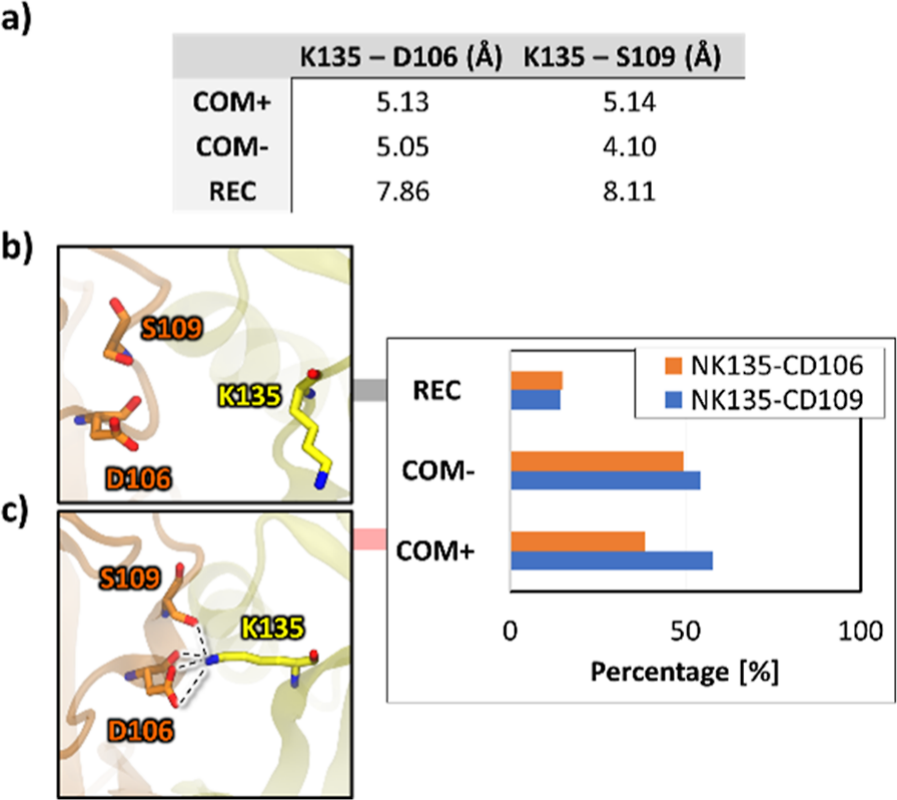
Distance comparison for a crucial contact between NKG2A-CD94 residues K135-D106 and K135-D109 in (b) **REC** and (c) **COM**^**+**^. The table in (a) summarized average distances, where lower values in **COM**^**+**^ and **COM**^**–**^ indicate a more frequent interaction when HLA-E is bound to the NKG2A/CD94 receptor. The histogram indicates the percentage of frames in which contact between residues is established, which is significantly higher in the case of **COM**^**+**^ and **COM**^**–**^. Representative snapshots in (b) **REC** where residues are not interacting and (b) **COM**^**+**^ where contact is established.

This observation is further supported with the distance analysis results, which show an increase of the average distance between pairs of residues in the absence of HLA-E with the values of 5.13, 5.05, and 7.86 Å for Lys_135_^NKG2A^–Asp_106_^CD94^ and 5.14, 4.10, and 8.11 Å for Lys_135_^NKG2A^–Ser_109_^CD94^, for **COM**^**+**^, **COM**^**–**^, and **REC**, respectively (Figure 8). This increase as well as variations in the distance between these residues for **REC** indicate less frequent interaction (Figures S19 and 20). In addition, the observed hydrogen bonds are likely the only new major interactions which are established after HLA-E binding between NKG2A and CD94 in the EC region, impacting linker regions. However, many energetically favorable contacts exist between HLA-E/peptide and NKG2A/CD94.

By visual examination of the interface between HLA-E and NKG2A/CD94 receptors, we identified further contacts in **COM**^**+**^ which may, according to our model, a complex network of hydrogen bonds fine-tuned by the ligand-specific nonameric peptide,^7,10^ play a role in signal transduction (Table S1). We observed that in the **COM**^**+**^ model, the nonameric peptide formed hydrogen bonds in Gln112^CD94^-peptide position (P)6 and Glu152^HLA–E^-P5 with CD94 and HLA-E (Figure S21). Additionally, Arg137^NKG2A^ was able to interact with Asp149^HLA–E^ in **COM**^**+**^ (Figure S22). The distance between residues was lower in **COM**^**+**^ than that in **COM**^**–**^, in which the two residues never interacted. Instead, Arg137^NKG2A^ in **COM**^**–**^ formed an H-bond with Glu152^HLA–E^ (Figure S23). This interaction was only possible due to the absence of a nonameric peptide. As Arg137^NKG2A^ is in close proximity to Lys135^NKG2A^ and can alter its positioning through backbone interactions, this may explain similar behavior of both **COM**^**+**^ and **COM**^**–**^ EC regions. Even more surprisingly, Arg137^NKG2A^ in **COM**^**–**^ also interacted with Ala150^HLA–E^, despite Ser151^HLA–E^ interacting similarly in both models (Figure S24).

Our simulations included the **COM**^**+**^ immune complex with the G form of the nonameric peptide that is able to successfully protect the target cell against the NK cells and the **REC** apo form of the complex with no peptide. MM/GBSA binding free-energy calculations revealed that the nonameric peptide provided marginally more favorable energies compared to **COM**^**–**^, which is comparable to previous studies.^10^ Namely, the interaction energy between the peptide and HLA-E/NKG2A/CD94 for **COM**^**+**^ was −105.55 ± 5 kcal/mol, which is consistent with previous computational studies of the complex. Subsequent per residue free-energy decomposition suggested that peptide residues 2, 8, and 9 had the largest favorable energy contributions to the complex (Table S2), which is in line with the studies of the truncated model of the complex.^7,10^ Between NKG2A/CD94 and the ligand, the energy was −53.0 ± 3 kcal/mol in **COM**^**+**^ and −33.71 ± 15 kcal/mol in **COM**^**–**^, showcasing a favorable energetic contribution of the peptide to the stability of the entire complex.

Additionally, investigation of the interaction energies between NKG2A and CD94 in the TM and IC domains revealed more favorable energies in **COM**^**+**^ and **COM**^**–**^ simulations than that in **REC** (Table S3). IC and TM energies were 10 kcal/mol lower in models with the bound HLA-E ligand, suggesting the formation of favorable interactions as a result of domain reorganization. More favorable energies were also noted in the linker regions with 3 and 8 kcal/mol lower energies for **COM**^**+**^ and **COM**^**–**^, respectively.

## Discussion

The inhibitory NKG2A/CD94 receptor is an important component on the surface of NK cells that enables the precise regulation of the cytotoxic response. The structure of the complete receptor is still unknown, and molecular events associated with inhibitory signal transduction to the IC ITIM region upon the CD94/NKG2A inhibitory receptor binding are largely unexplored.

Models of the immune receptor complexes in this study were constructed based on the available crystal structure of the immune receptor complex (PDB id: 3CDG),^23^ featuring the full extent of the interactions between the NK receptor NKG2A/CD94 and its ligand HLA-E. This ligand presents a nonameric G peptide, which plays a key role in establishing important contacts with the receptor and therefore allowing a signal to be transmitted across the membrane.^7^ As the structures of the TM and cytoplasmic domains of the receptor have not yet been resolved experimentally, the complete immune complex first had to be generated. An NMR structure of the TM receptor NKG2C in complex with DAP12 has been determined,^63^ yet the sequence similarity in the TM region between the two receptors is only 28.9%, which was deemed insufficient to produce an accurate homology model of NKG2A. The best alignment for the CD94 protein amounted to only a 15.38% sequence identity with the best-aligned sequence from the 6T15 PDB structure.^64^ Furthermore, NKG2A forms dimeric interactions with CD94 in the membrane region, which requires the determination of suitable orientations between helices. However, AI for structure prediction has made significant advances in protein 3D structure prediction, including protein dimer structures, which allows for accurate structure approximation. Thus, we present here for the first time the complete model of this inhibitory receptor and its complex with the EC portion of the HLA-E ligand.

One of the interesting structural aspects of the predicted regions of the NKG2A/CD94 receptor was the presence of an α-helix in the place of the C-terminal ITIM region. Although a large helical structure possibly may not be native, we suspected that helix formation may serve an important role in signal transduction, as dynamical helix formation has been observed in similar receptors.^61^

A pressing concern with conducting large systems, all-atom simulations are ensuring a long enough simulation time to allow for exploration of the entire conformational space and analyzing only the equilibrated part of the trajectory.^65^ In our case, although the RMSD value was higher in **REC**, there were smaller changes in RMSD in **REC** than **COM**^**+**^ even in the equilibrated part of the trajectories. This is due to the dynamical nature of the complex as the protein linker regions in **COM**^**+**^ allowed for rotation of the receptor head and tilting of the EC regions. Similar behavior has been observed in other TM immune receptors such as TLR4, which exhibits a bouncing movement.^66^ The flexible nature of the receptor is further exemplified in the PCA analysis. We should emphasize that the conformational landscape available to the macromolecular system under study is vast and cannot currently be assessed in its entirety with available computational capabilities. In this respect, our work, based on 2 μs long simulations of the selected systems, should be understood as a modeling study supported by MD simulations to propose an atomistic model of the signal transduction process. This is consistent with similar attempts to unravel the key conformational and dynamic changes that lead to signal transduction.^54^

Next, we have assessed the range of structural and dynamical changes as a result of HLA-E binding to the NKG2A/CD94 receptor. This is the first domain through which a signal is transmitted, and we have quantified changes through both large-scale changes in protein dynamics and specific interactions that guide conformational change, which previous studies have also reported on.^7,10^ On a global scale, ligand binding slightly stabilized the ECD of NKG2A and CD94 and induced correlations between linker and TM regions, supported by results from essential dynamics and RMSF analysis. The eigenvectors revealed a pronounced change in the dynamics of the ICD of NKG2A, accompanied by higher flexibility upon ligand binding.

The main effect of signal transduction is reflected in the ITIM domains of NKG2A. These domains play a key role in the transduction of the inhibitory signal by providing a binding site for SHP phosphorylases.^67^ Based on our simulations, we propose that the difference in the distance between ITIMs in the presence of bound HLA-E may be an indicator of key changes in the environment of ITIM Tyr8 and Tyr40, which are phosphorylated by the appropriate tyrosine kinases. Together with the RDF analysis, the results suggest that ITIM side-chain oxygen atoms of these two tyrosines become more exposed while HLA-E is bound to the receptor, possibly due to a larger distance from the lipid bilayer.

Our data further suggest that the TM helices of NKG2A and CD94 position differently in HLA-bound (crossed shape) vs unbound models (Y shape), which would allow for signal propagation through the membrane. Specific interactions in the membrane proximal ICD were noted, which were possible only through correct helix positioning in the TMD. As specific interactions formed simultaneously in the ICD and ECD, this may confirm the findings that rearrangements in the juxtamembrane regions could be crucial for receptor activation. This was supported by energy calculations, which revealed that the energy between NKG2A and CD94 subunits was more favorable in all regions after HLA-E binding, particularly in the TMD and ICD. These results are in accordance with the growing evidence that the change in helix positioning in the TMD is crucial for signal transduction in single-pass TM receptors. Namely, the change in crossing angles between the two helices causes changes in the distance between the protruding intracellular parts, which in term alters their intracellular structure.^68^

We also investigated whether there is a difference in the strength of specific interactions between models with HLA-E and the apo form of the receptor. According to previous computational studies, large-scale internal changes in the ECD are unlikely. Instead, intricate contacts which guide signal transduction were proposed. We found that the formation of the interactions between the Lys_135_^NKG2A^–Asp_106_^CD94^ and Lys_135_^NKG2A^–Ser_109_^CD94^ might be a key requirement for signal transduction. In the case of the apo form of the receptor, the residues required for this interaction were not suitably positioned for hydrogen bond formation. In the case of the hypothetical peptide-less **COM**^**–**^ form of the complex, additional newly discovered interactions between Arg137^NKG2A^ and HLA-E might still enable proper residue positioning for signal transduction as these interactions still stabilize the receptor and enable linker reorganization despite interacting with different residues from HLA-E. This augments our knowledge about the flexibility of signal transduction and the role of the peptide in the complex as changes in the receptor structure in its absence still indicated that moderate transduction could occur.

**Figure 9:**
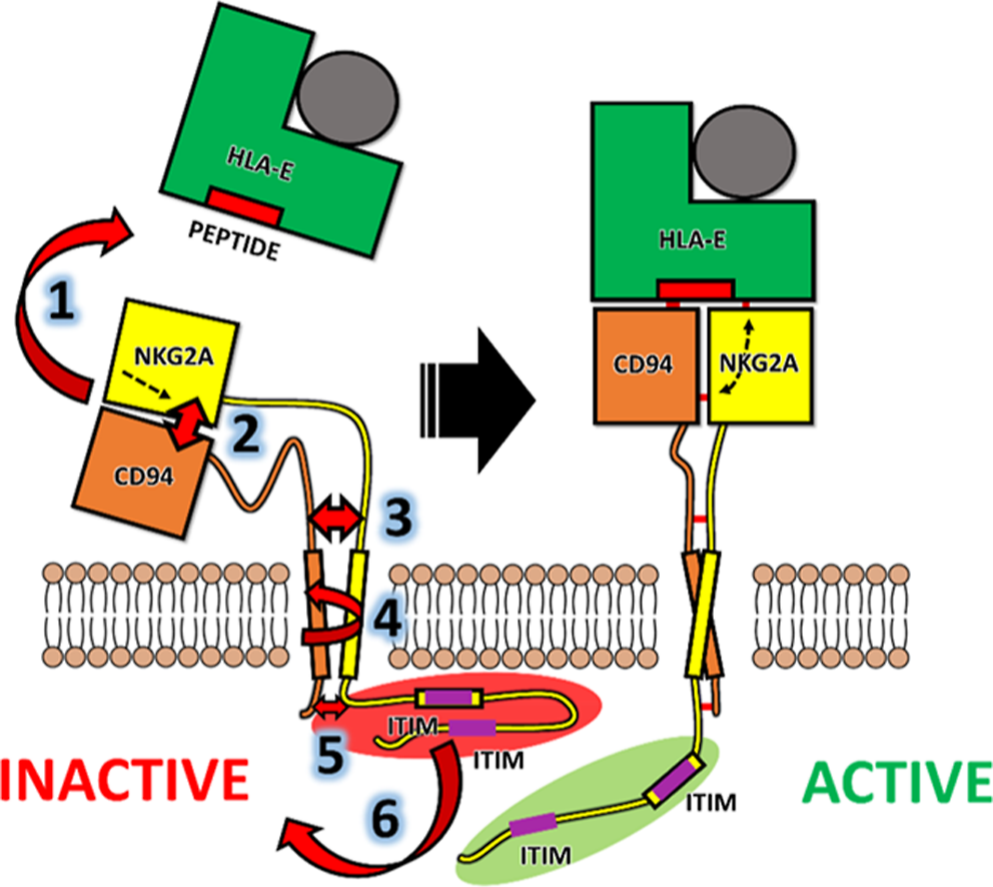
Key events of the proposed inhibitory signal transduction after HLA-E binding to the NKG2A/CD94 receptor based on the performed all-atom molecular simulations: (1) binding of HLA-E. (2) Formation of key hydrogen bonds Lys_135_^NKG2A^–Asp_106_^CD94^ and Lys_135_^NKG2A^–Ser_109_^CD94^. (3) Linker reorganization, (4) Repositioning of TM helices. (5) IC interaction between NKG2A and CD94. (6) ITIM repositioning.

Based on the reported findings from the performed simulations, we propose a model of events for NKG2A/CD94 signal transduction through HLA-E binding (Figure 9). (1) Binding of the HLA-E ligand establishes the interactions between the NKG2A/CD94 receptor and the nonameric peptide, as well as HLA-E. (2) This hydrogen-bonded network reorganizes the key residues in NKG2A/CD94, which induces the formation of hydrogen bonds Lys_135_^NKG2A^–Asp_106_^CD94^ and Lys_135_^NKG2A^–Ser_109_^CD94^. This enables greater communication between each of the receptor head parts (CD94 and NKG2A) and locks the protein complex in a different set of conformations. (3) This then allows the linker regions between NKG2A and CD94 to position for favorable hydrogen bonding (Ser34^CD94^–Thr96^NKG2A^) and for the reorganization of linker regions, which interact more favorably. However, the reorganization of the linker region is probably not a direct consequence of the hydrogen bonds formed in the ECD since long and flexible linker parts exist between these two structural components of the receptor. It is also influenced by the upright positioning of the ECD after HLA-E binding. (4) The signal is then transmitted through the lipid bilayer through the repositioning of TM helices, which may adopt a more crossed structure after HLA-E binding. (5) The angle at which the helix protrudes from the membrane allows the formation of stable interactions between the five-residue intracellular chain of CD94 with the flexible membrane proximal NKG2A chain. (6) Ultimately, this affects the positioning of both ITIMs, as well as their exposure to the solvent molecules. As ITIM phosphorylation is required for the binding of the protein tyrosine phosphatases SHP-1 and SHP-2, greater accessibility of ITIMs is likely a major reason for the successful transduction of the inhibitory signal.

As many ITIM-bearing receptors such as KIR, PECAM-1, and CD72 have a similar intracellular structure and function,^18^ this type of signal transduction might be common elsewhere as well. In this case, the C-terminal ITIM is the first candidate for phosphorylation to take place, after which the N-terminal ITIM follows suit.

In addition, the interactions with the lipid bilayer may also play an important role for successful signal transduction. The greatest impact of the membrane on signal transduction is likely that it promotes linker reorganization.^69^ Our contact analysis revealed that when NKG2A/CD94 interacts with the membrane in the HLA-E-unbound state, the linkers adopt different random conformations with a smaller number of interchain interactions. From the beginning, two cells are linked via CD94/NKG2A and HLA-E. It should be pointed out that the relative orientation between the NK and target cell with the HLA-E ligand when engaging with the receptor is also an important factor that unfortunately could not be taken into account in this study.

Due to the absence of CD94/NKG2A interactions in the IC region when HLA-E is not bound, the membrane-proximal NKG2A residues can form interactions with the charged groups of the lipid bilayer, leading to a conformation which is unsuitable for the phosphorylation of ITIMs and subsequent binding of SHP phosphatases. Membrane interactions are thus implicated both in the dynamics and organization of EC parts of the protein, as well as in the positioning of ITIM regions.^61^

In our simulations, we have tried to construct the molecular systems in as much detail as possible; however, there may be other subtle factors involved in the transduction of the inhibitory signal. For example, understanding the changes in receptor behavior as a result of membrane composition changes and interactions with activating receptors, which are still not clear, would be required to paint a fuller picture on immune receptor signaling. Additionally, mapping large-scale changes in protein behavior and investigating the entire conformational space of the protein complex would reveal even more intricacies in TM signal transduction.

## Conclusions

With the aid of the Alphafold 2 artificial intelligence system, we have constructed the first 3D model of the complete NKG2A/CD94 receptor and its corresponding immune complex with HLA-E ligand and evaluated it in extensive all-atom MD simulations. The models showed that the interplay of events taking place between the EC and TM regions ultimately affect the ITIM regions, which comprise the point where the signal is transmitted further down the inhibitory signaling cascade, ultimately protecting the target cell against the cytotoxic effects of NK cells. The research we have performed provides new atomistic details of the fundamental NK cell protection mechanism that may guide and inspire succeeding structural and biochemical experiments. In addition, structure-based foundation is now provided for the utilization of the NKG2A/CD94/HLA-E/β-2m/peptide immune complex in drug discovery against diverse pathologies including viral infections, cancer, and the elimination of senescent cells, a potential new therapeutic approach for many age-related diseases.

## Data Availability

All molecular simulations, analysis, and visualization were performed with widely used programs available freely for academic institutions: Gromacs 2019, Amber20 and AmberTools20, VMD 1.9.3, PyMol 2.0, and ChimeraX. All procedures and workflows are described in the Methods section. Structure and parameter files are provided in the Supporting Information. Additional data including input files and final structures are available at Zenodo: doi.org/10.5281/zenodo.7524705.

## Supporting Information Available

Protein sequence and domain naming; structure prediction images and graphs; RMSD, correlation matrices, and PCA graphs; IC analysis graphs and TM and linker analysis graphs; ECD analysis graphs; ECD hydrogen bond visualization; summarized hydrogen bond persistence and distance; and MMGBSA energy calculation (PDF)
Trajectories of two models, structure file of the complex, and topology (ZIP)
