# Supplementary material for: All-Atom Simulations Reveal the Intricacies of Signal Transduction upon Binding of the HLA-E Ligand to the Transmembrane Inhibitory CD94/NKG2A Receptor
Source: J Chem Inf Model. 2023 May 19;63(11):3486–99. doi: 10.1021/acs.jcim.3c00249 (PMC10268964; doi:10.1021/acs.jcim.3c00249)
Supplement: Supplementary file 1 — ci3c00249_si_001.pdf [file ci3c00249_si_001.pdf]

SUPPORTING INFORMATION

All-atom simulations reveal the intricacies of signal transduction upon binding of HLA-E ligand to the transmembrane inhibitory CD94/NKG2A receptor

Martin Ljubič[†,&], Eva Prašnikar[†], Andrej Perdih[†,&], Jure Borišek[†,*]

[†]National Institute of Chemistry, Hajdrihova 19, 1000, Ljubljana, Slovenia

[&]Faculty of Pharmacy, University of Ljubljana, Aškerčeva 7, 1000 Ljubljana Slovenia

Corresponding author*:

E-mail: jure.borisek@ki.si

Table of contents

Supporting figures

Figure S1 3
Figure S2 4
Figure S3 5
Figure S4 6
Figure S5 7
Figure S6 8
Figure S7 9
Figure S8 10
Figure S9 11
Figure S10 12
Figure S11 13
Figure S12 14
Figure S13 15
Figure S14 16
Figure S15 17
Figure S16 18
Figure S17 19
Figure S18 20
Figure S19 21
Figure S20 22
Figure S21 23
Figure S22 24
Figure S23 25
Figure S24 26

Supporting tables

Table S1 28
Table S2 29
Table S3 30

Supporting movies

Movie S1 31

Supporting figures

a)

1 MDNQGVIYSDLNLPPNPKRQQRKPKGNKNSILATEQEITYAELNLQKASQ 50
51 DFQGNDKTYHCKDLPSAPEKLIVGILGIICLILMASVVTIVVIPSTLIQR 100
101 HNNSSLNTRTQKARHCGHCPEEWITYSNSCYYIGKERRTWEESLLACTSK 150
151 NSSLLSIDNEEEMKFLSIISPSSWIGVFRNSSHHPWVTMNGLAFKHEIKD 200
201 SDNAELNCAVLQVNRLKSAQCGSSIIYHCKHK 232

1 MAVFKTTLWRLISGTLGIICLSLMSTLGILLKNSFTKLSIEPAFTPGPNI 50
51 ELQKDSDCCSCQEKWVGYRCNCYFISSEQKTWNESRHLCASQKSSLLQLQ 100
101 NTDELDFMSSSQQFYWIGLSYSEEHTAWLWENGSALSYLFPSFETFNTKN 150
151 CIAYNPNGNALDESCEDKNRYICKQQLI 179

1 SHSLKYFHTSVSRPGRGEPRFISVGYVDDTQFVRFDNDAASPRMVPRAPW 50
51 MEQEGSEYWDRETRSARDTAQIFRVNLRTLRGYYNQSEAGSHTLQWMHGC 100
101 ELGPDRRFLRGYEQFAYDGKDYLTLNEDLRSWTAVDTAAQISEQKSNDAS 150
151 EAEHQRAYLEDTCVEWLHKYLEKGKETLLHLEPPKTHVTHHPISDHEATL 200
201 RCWALGFYPAEITLTWQQDGEGHTQDTELVETRPAGDGTFQKWAAVVVPS 250
252 GEEQRYTCHVQHEGLPEPVTLRW 273

1 MIQRTPKIQVYSRHPAENGKSNFLNCYVSGFHPSDIEVDLLKNGERIEKV 50
51 EHSDLSFSKDWSFYLLYYTEFTPTEKDEYACRVNHVTLSQPKIVKWDRDM 100

1 VMAPRTLFL 9

b)

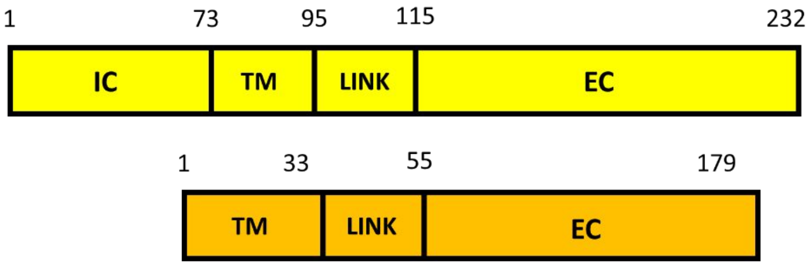

Figure S1. Sequence and naming of the entire NKG2A/CD94/HLA-E protein complex used in the simulation. a) Sequence of NKG2A (yellow), CD94 (orange), HLA-E (green), β-2m (gray) and nonameric peptide (red). b) Subdivision of NKG2A (yellow): IC, TM, LINK and EC regions. Subdivision of CD94 (orange): TM, LINK and EC regions.

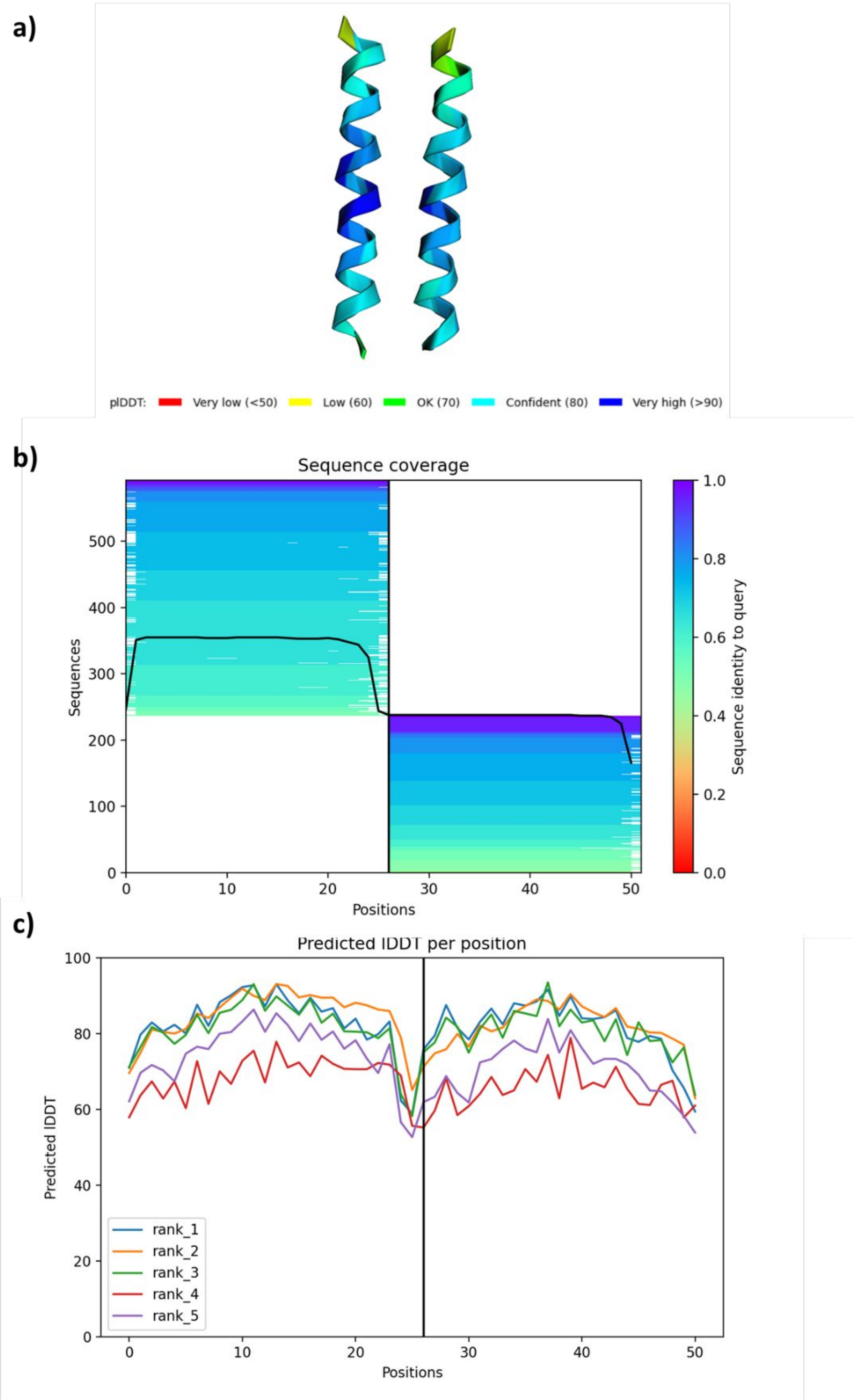

Figure S2. Best NKG2A/CD94 transmembrane dimer structure, predicted by Colabfold. a) pIDDT (Predicted Local Distance Difference Test), projected onto the structure of the NKG2A/CD94 transmembrane dimer b) Sequence coverage graph, showcasing the number of homologous sequences found for each helix c) pIDDT graph for the top 5 ranked models.

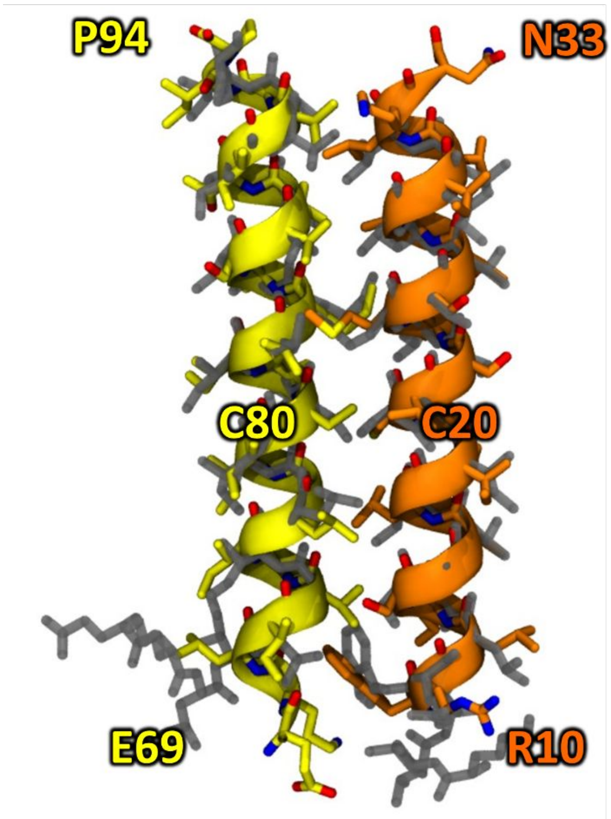

Figure S3. Comparison of the Colabfold generated model with homology models. The NKG2A (yellow) and CD94 (orange) dimer was generated using Colabfold. Homology models were aligned to the structure and are shown in gray. The backbone and most sidechains adopt a similar conformation, apart from the sidechains of Glu69^{NKG2A} and Arg10^{CD94}, which position differently. Residues Cys80^{NKG2A} and Cys20^{CD94} have been marked.

a)

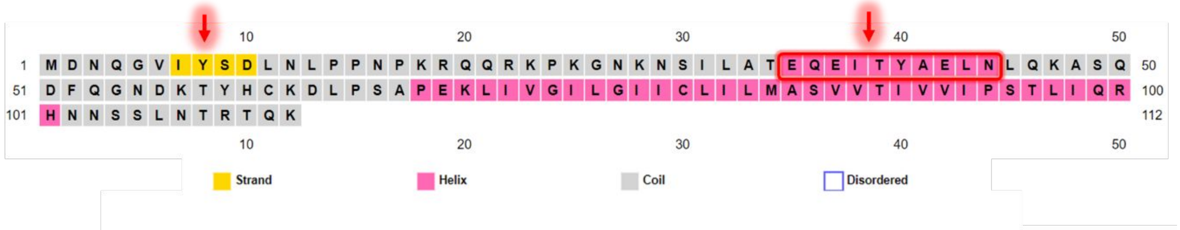

b)

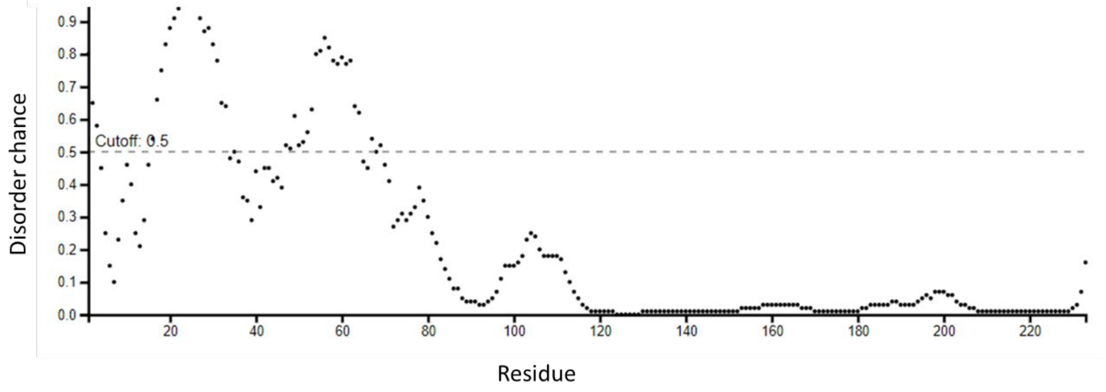

Figure S4. Prediction of the NKG2A structure. a**)** PSIPRED4 secondary structure prediction of the NKG2A intracellular region. ITIM tyrosine residues are indicated with a red arrow and a possible α-helix region has been circled. b) DISOPRED prediction, indicating the probability of disorder for each residue. A dip in disorder probability is seen around residue 40, indicating possible ordered segments at the position of the C-terminal ITIM.

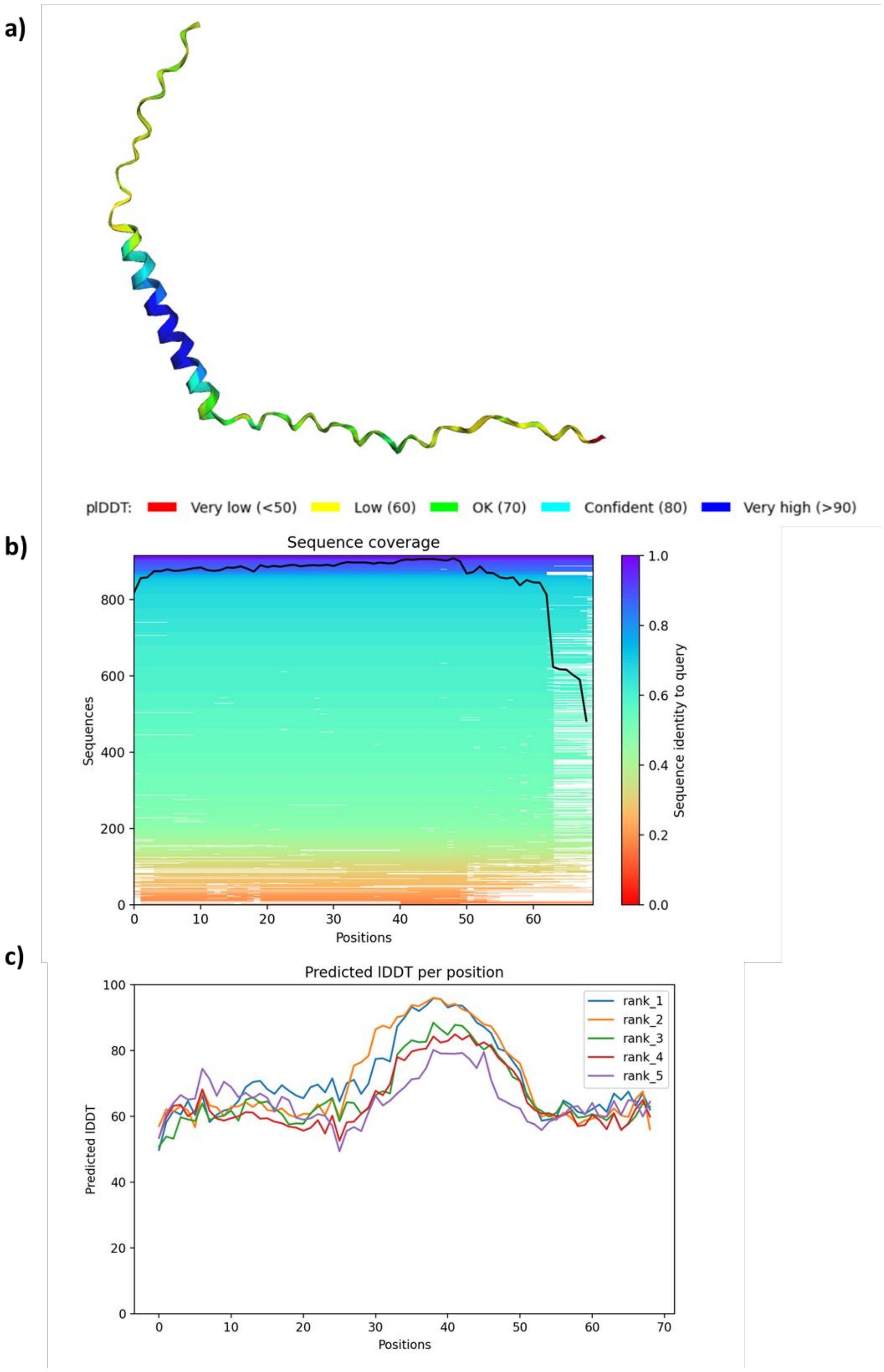

Figure S5. Best NKG2A intracellular region structure, predicted by Colabfold. a) pIDDT, projected onto the structure b) Sequence coverage graph, showcasing the number of homologous sequences found c) pIDDT graph for the top 5 ranked models.

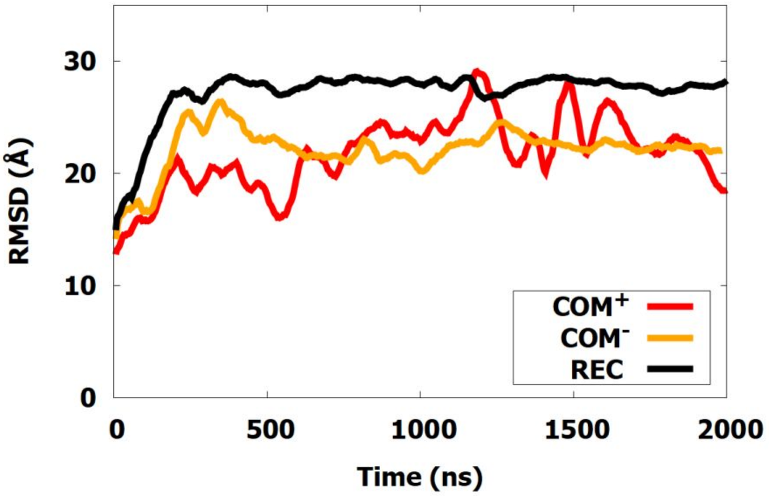

Figure S6. Root Mean Square Deviation (RMSD) values of the **COM⁺** (red), **COM⁻** (orange) and **REC** (black) models vs. simulation time. Only the receptor part of the **COM⁺** and **COM⁻** models was used in the alignment and calculation of RMSD values. A moving average of 15 was used to display the results.

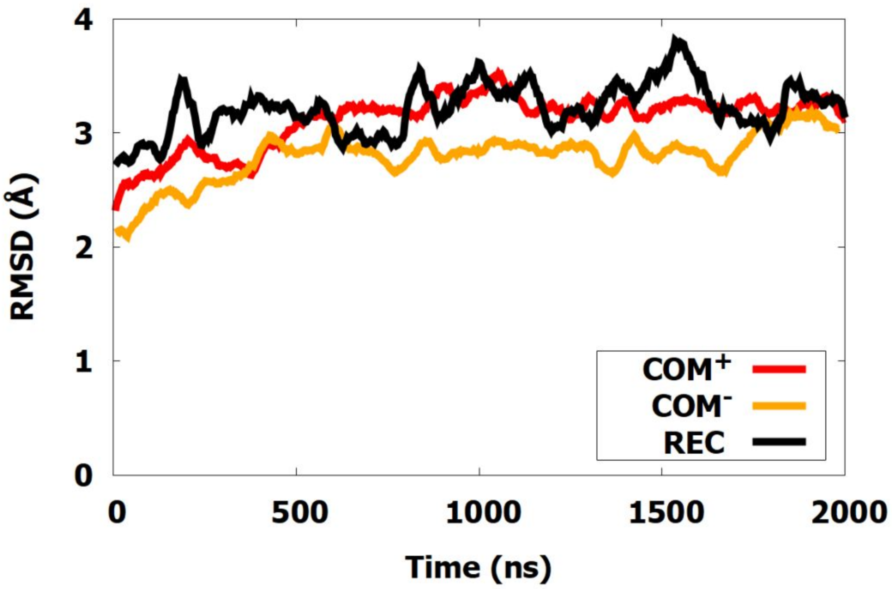

Figure S7. Root Mean Square Deviation (RMSD) values of the **COM$^+$** (red), **COM$^-$** (orange) and **REC** (black) models vs. simulation time for the ECD of NKG2A/CD94. Only the ECD of the models was used in the alignment and calculation of RMSD values. A moving average of 15 was used to display the results.

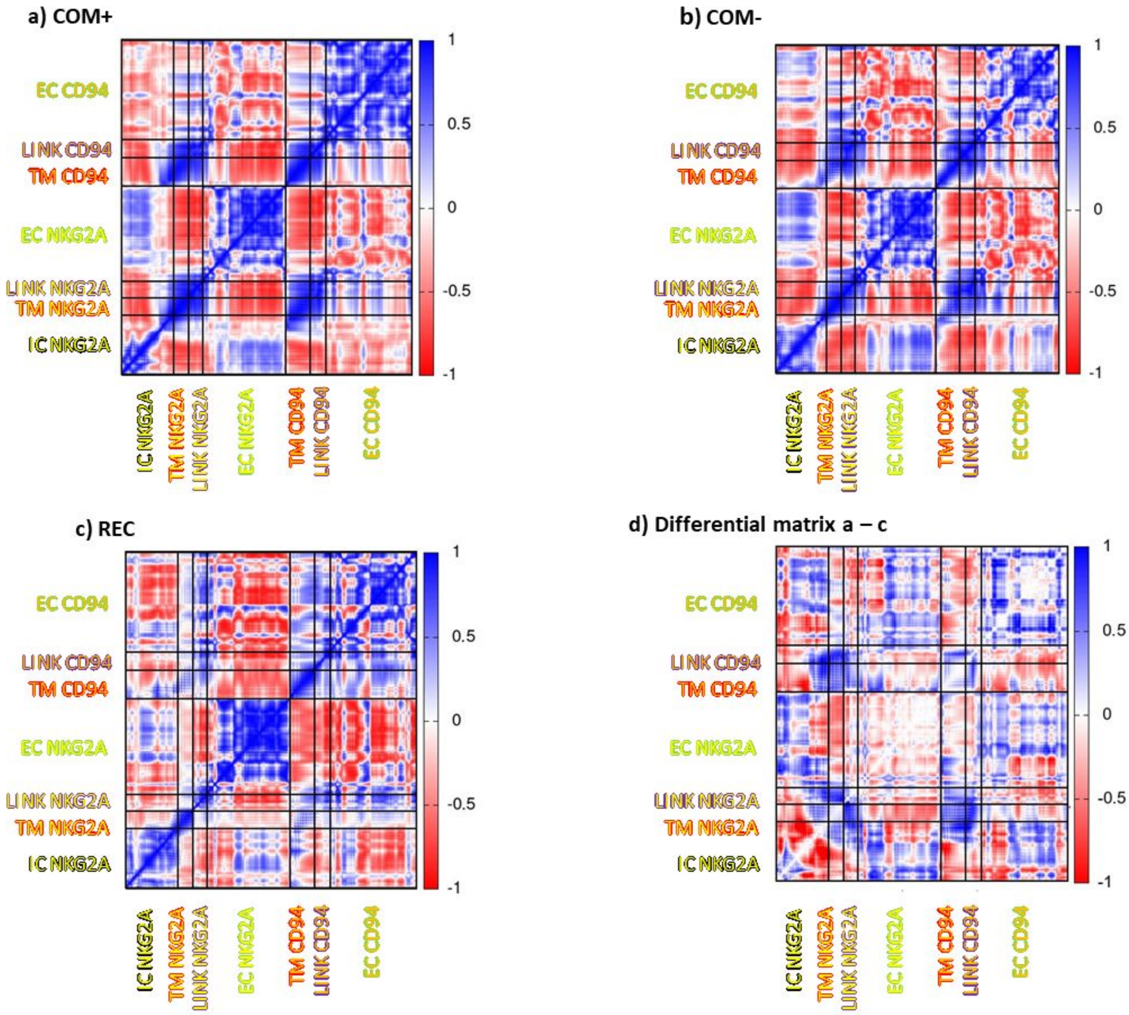

Figure S8. Cross-correlation matrices for models a) **COM⁺**, b) **COM-**, c) **REC** and d) differential matrix, calculated as a difference between models COM+ and REC.

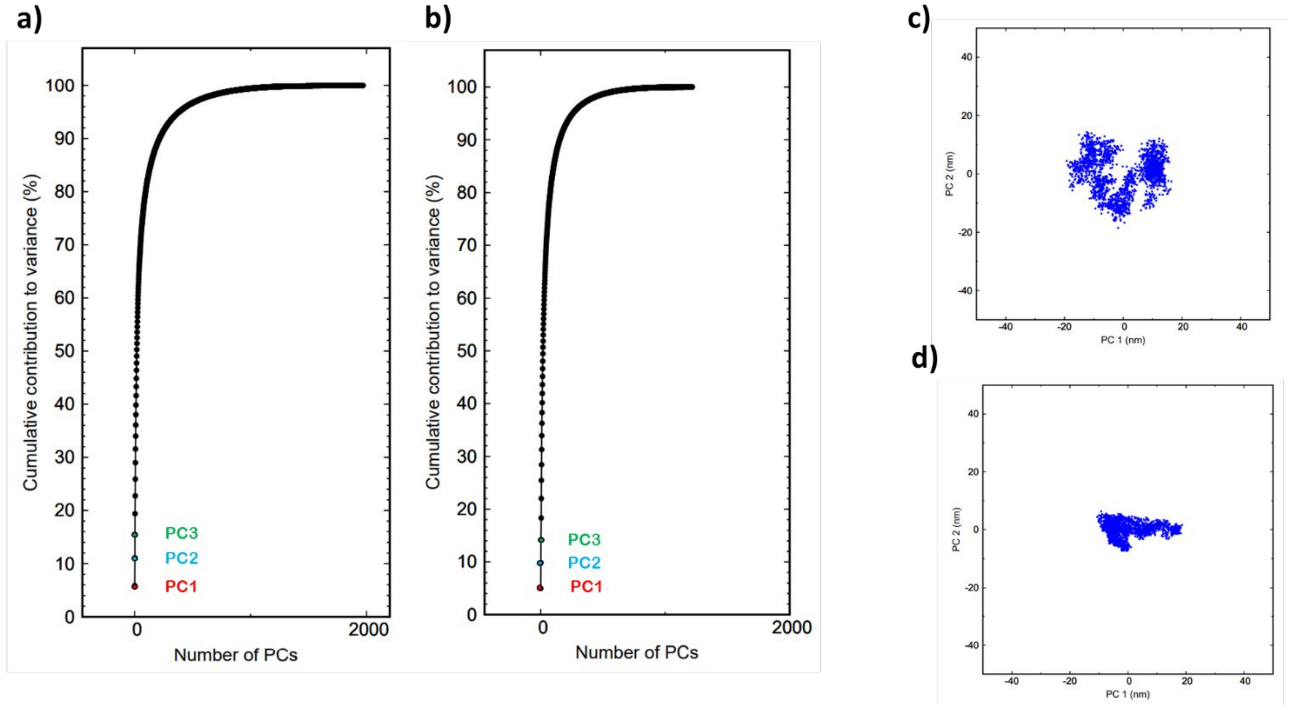

Figure S9. Principal components analysis (PCA) cumulative contribution to variance for: a) **COM⁺** and b) **REC**. On y-axis is depicted Cumulative contribution of PCs (x-axis) to the variance of the overall motion calculated upon Principal Component Analysis. The contributions from the first three PCs are highlighted in red, blue and green, respectively. Scatter plots for: c) **COM⁺** and d) **REC** models. Scatter plots represent the projections of the Cα and P atoms displacements along the trajectory onto the first principal component (PC1, x-axis) vs. the projections onto the second principal component (PC2, y-axis). The PC cumulative contribution showed that the first two PCs accounted for 10% of the variance to the global protein motion in both the **COM⁺** and **REC** models. This obtained value is relatively small, but is likely the result of the large size of the membrane-embedded protein complex relative to the simulation time, further exemplified by the shape of the scatterplots.

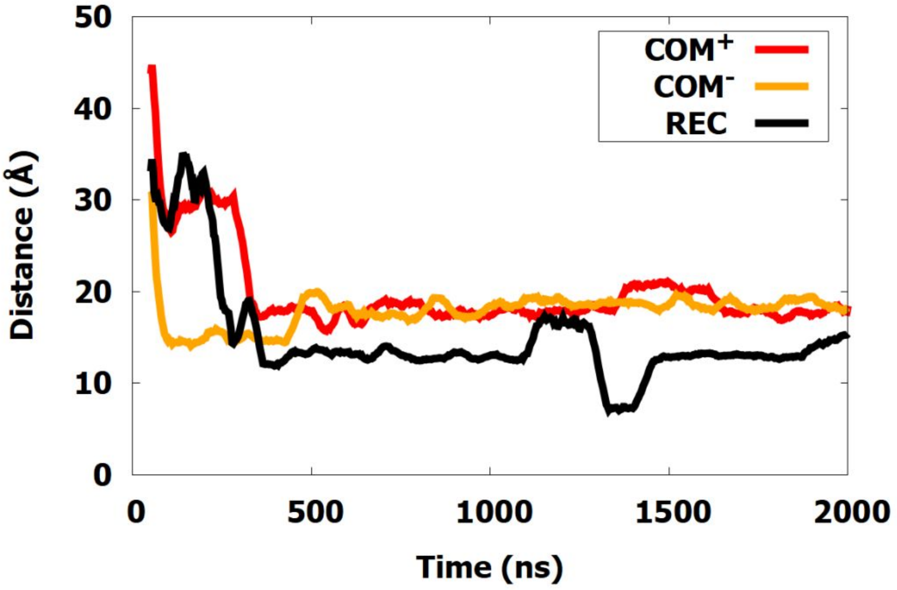

Figure S10. Distances between OH@Tyr8 and OH@Tyr40 of NKG2A for models **COM**$^{+}$ (red), **COM-** (orange) and **REC (**black).

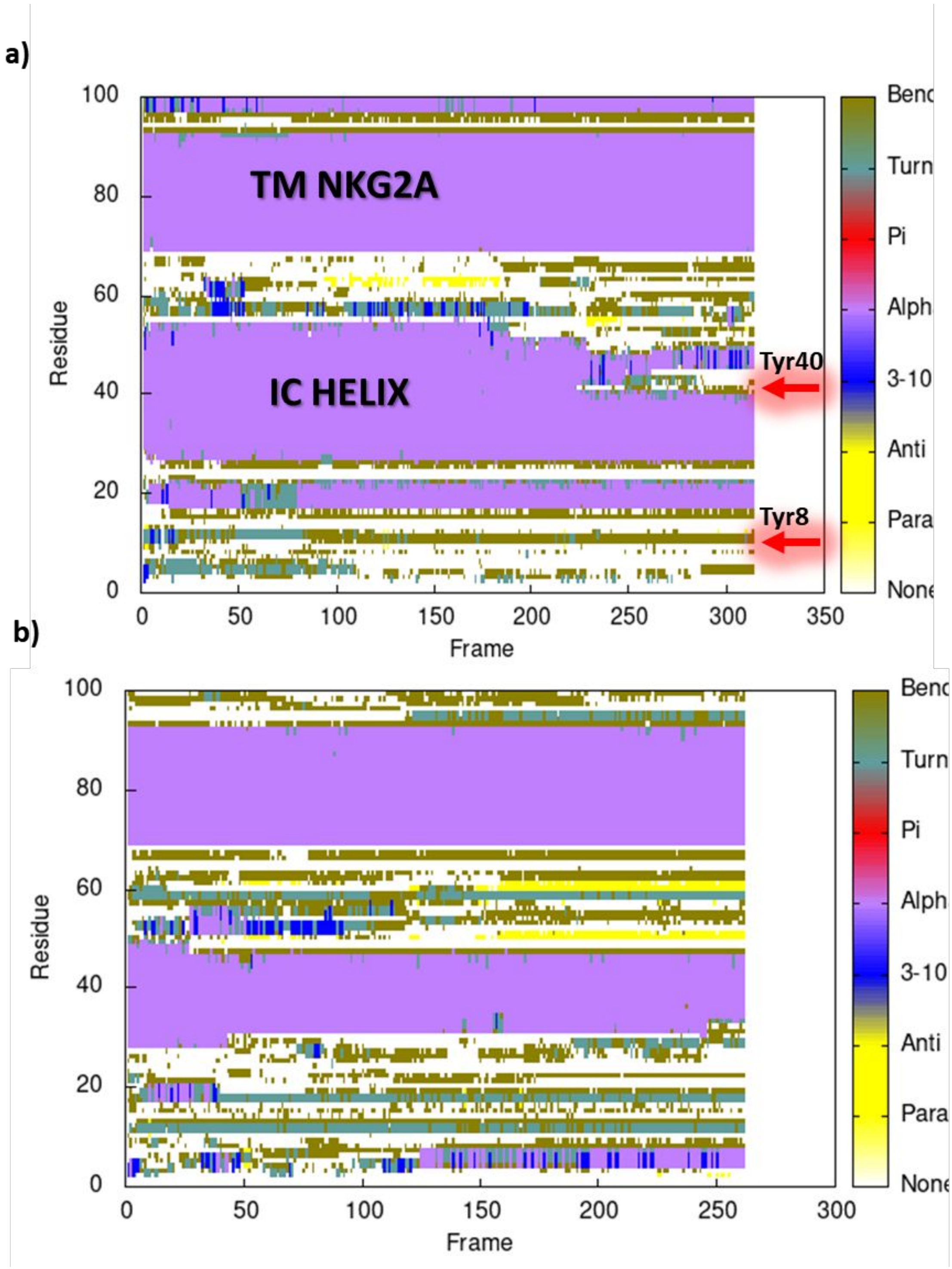

Figure S11. DSSP plots of residues 1 to 100 of NKG2A protein for a) **COM⁺** and b) **REC**. Red arrows indicate tyrosine Tyr8 and Tyr40 residues of ITIM regions. Colors in each frame represent different types of secondary structure: α-helix (purple), 3-10 helix (blue), π-helix (red), β-sheet (yellow), bend (brown), turn (cyan) and unstructured (white).

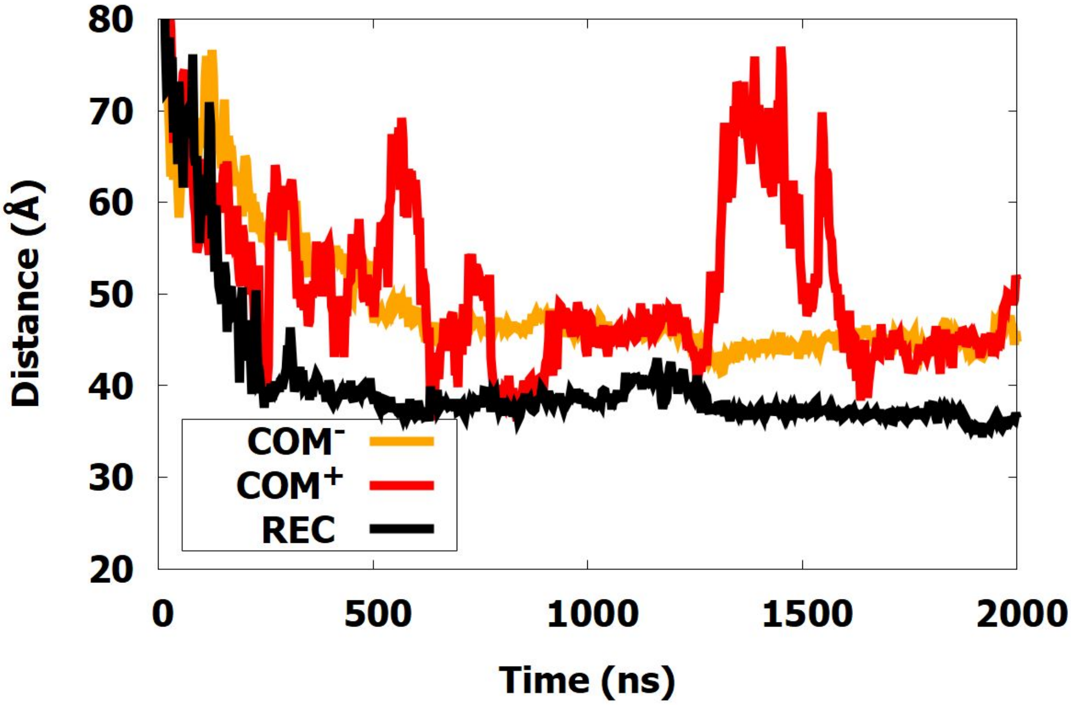

Figure S12. Distances between the center of mass for **COM⁺** (red), **COM-** (orange) and **REC** (black) of residues 1-45 of NKG2A and the TM regions of NKG2A/CD94.

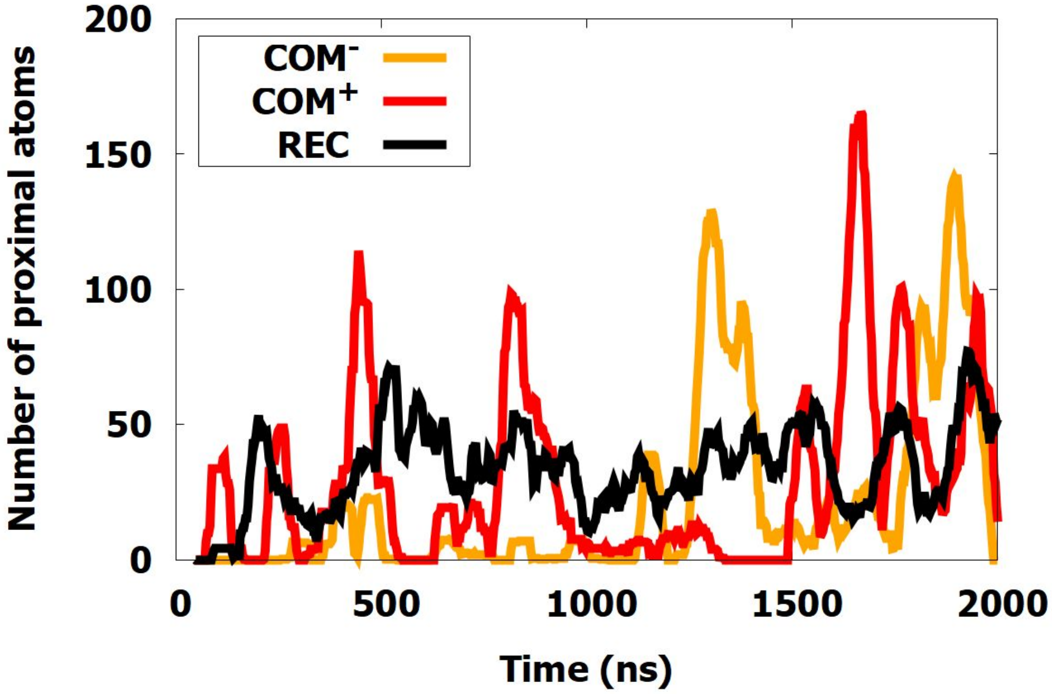

Figure S13. Total number of proximal atomic contact pairs between the IC 1-45 region of NKG2A/CD94 and membrane lipids in **COM**$^{+}$ (red), **COM**$^{-}$ (orange) and **REC** (black). The cutoff distance was set to 4 Å and a moving average of 15 was used for data processing.

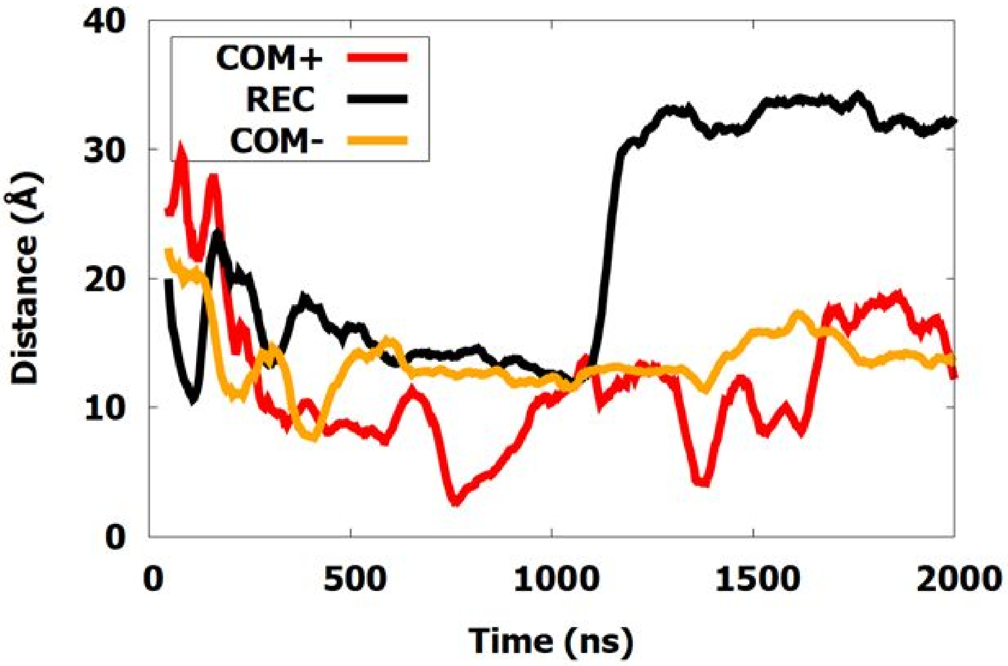

Figure S14. Distances between the center of mass for **COM⁺** (red), **COM-** (orange) and **REC** (black) of residues 1-3 of the CD94 protein and residues 53-63 of NKG2A protein.

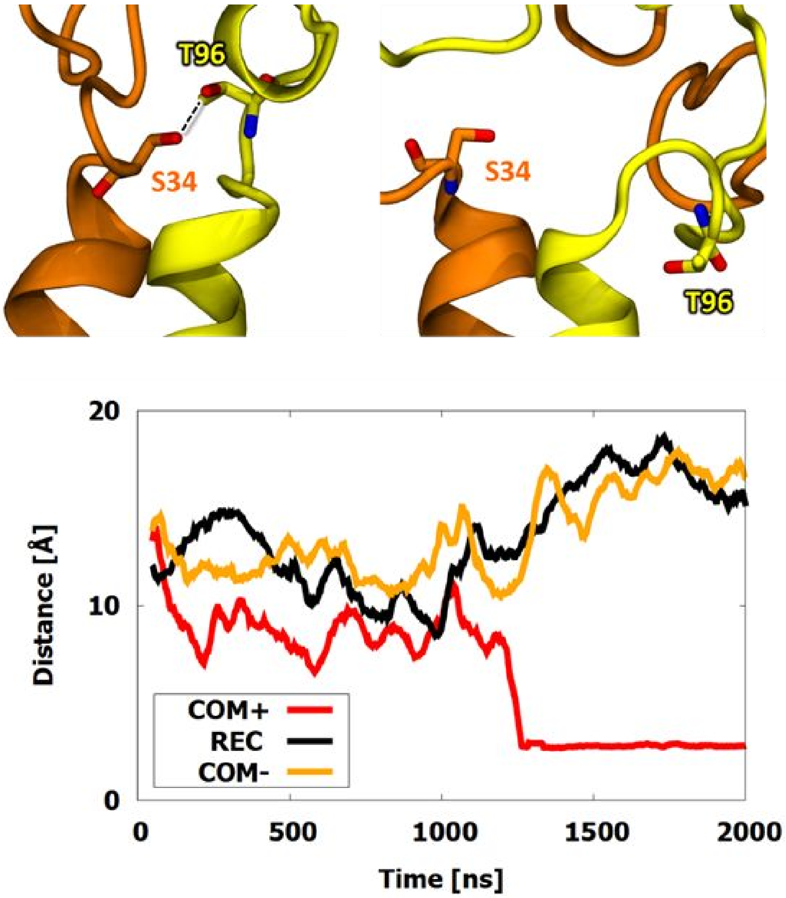

Figure S15. Hydrogen bond formation between OG@Ser34^{CD94} and OG@Thr96^{NKG2A}, located at the top of the transmembrane region. a) Image of the interaction in **COM**$^{+}$ at 2 µs (left) and the lack of an interaction in **REC** at 2 µs (right). b) Distance between atoms for **COM**$^{+}$ (red), **COM-** (orange) and **REC** (black) as a function of simulation time.

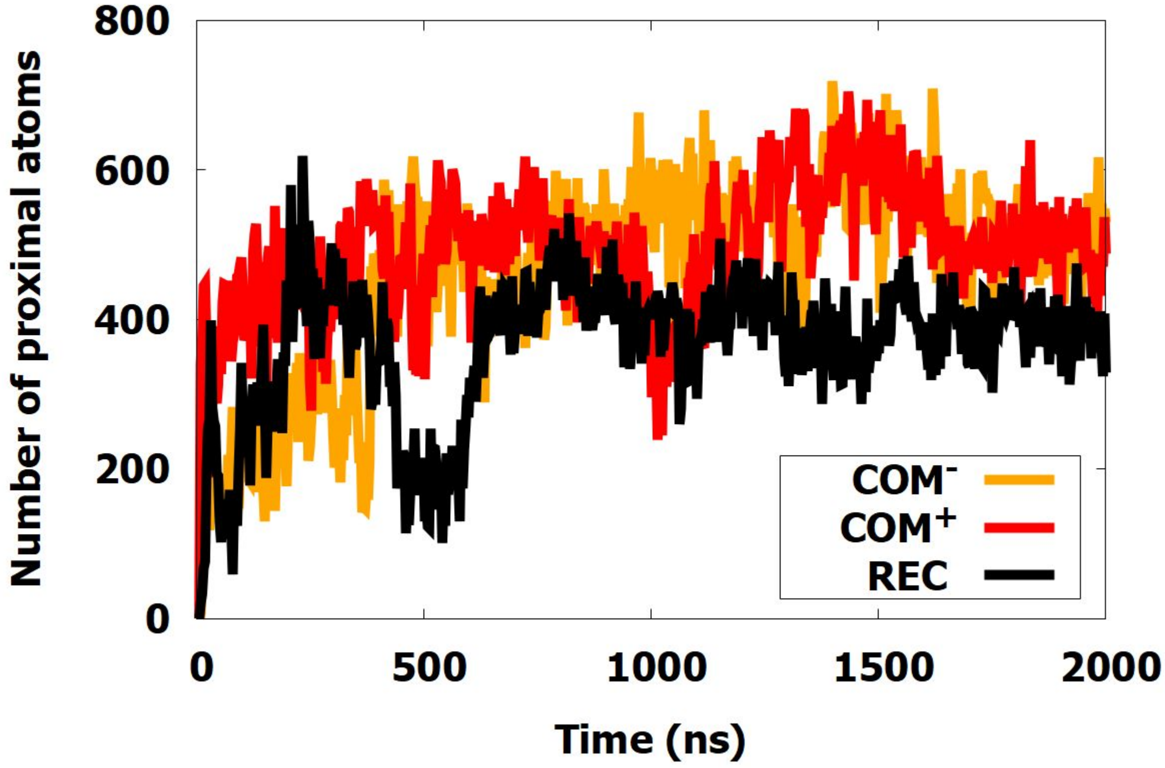

Figure S16. Total number of proximal atomic contact pairs between the linker regions of NKG2A and CD94 in **COM**⁺ (red) and **REC** (black) The cutoff distance was set to 4Å.

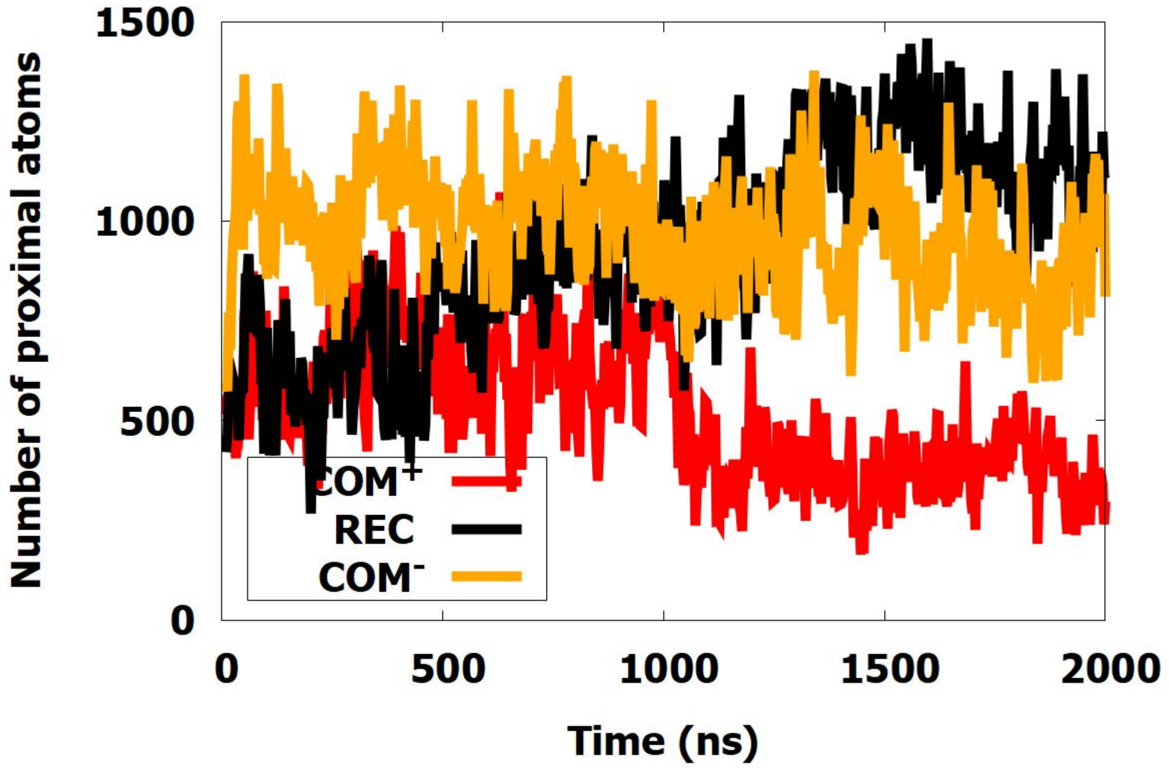

Figure S17. Total number of proximal atomic contact pairs between the linker regions of NKG2A and CD94 and membrane lipids in **COM**$^+$ (red), **COM**$^-$ (orange) and **REC** (black) The cutoff distance was set to 4Å.

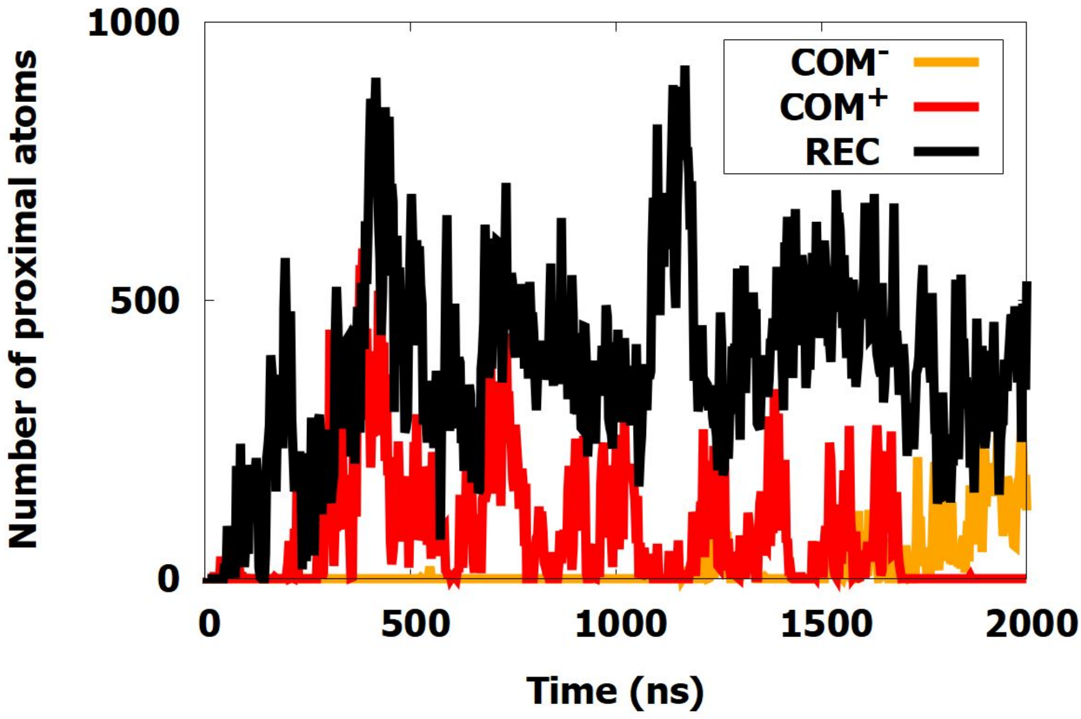

Figure S18. Total number of proximal atomic contact pairs between the extracellular domain (ECD) of NKG2A/CD94 and membrane lipids in **COM**$^{+}$ (red) and **REC** (black). The cutoff distance was set to 4Å.

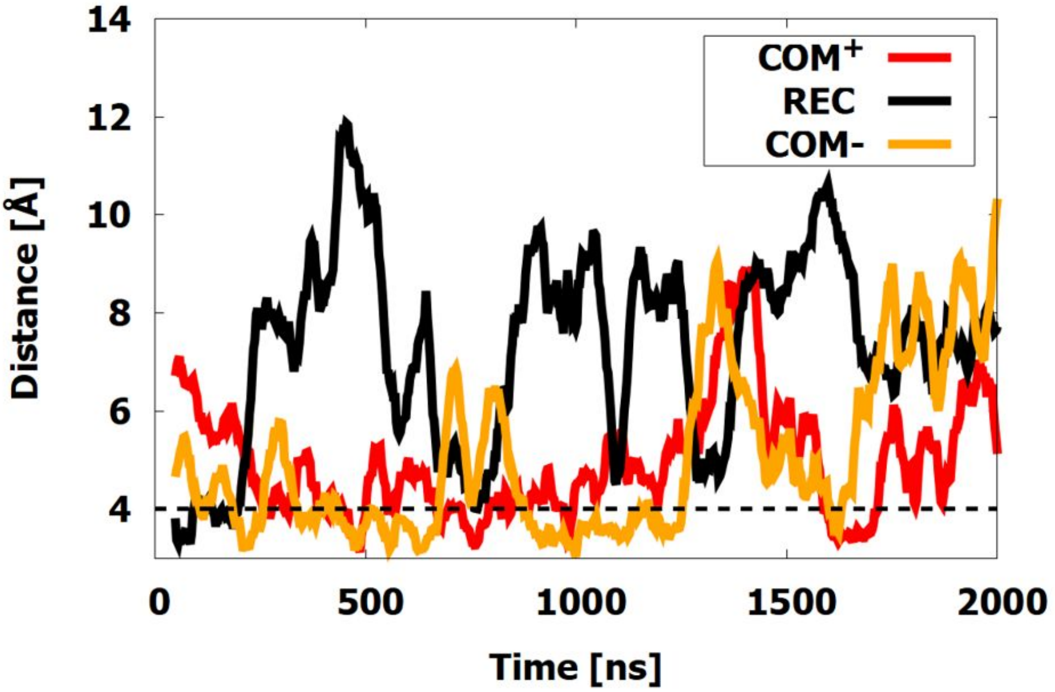

Figure S19. Distances between atoms O@Asp106 of CD94 and NZ@Lys135 of NKG2A vs. simulation time for models **COM+** (red), **COM-** (orange) and **REC** (black). The moving average with the interval 15 was used for data processing.

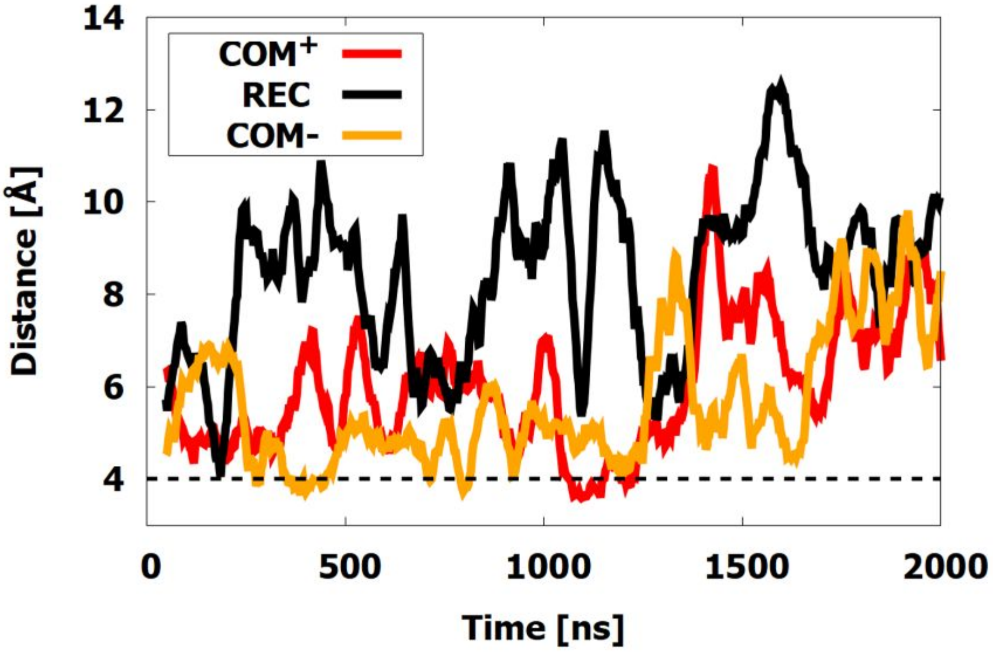

Figure S20. Distances between atoms OG@Ser109 of CD94 and NZ@Lys135 of NKG2A vs. simulation time for models **COM+** (red), **COM-** (orange) and **REC** (black). The moving average the with interval 15 was used for data processing.

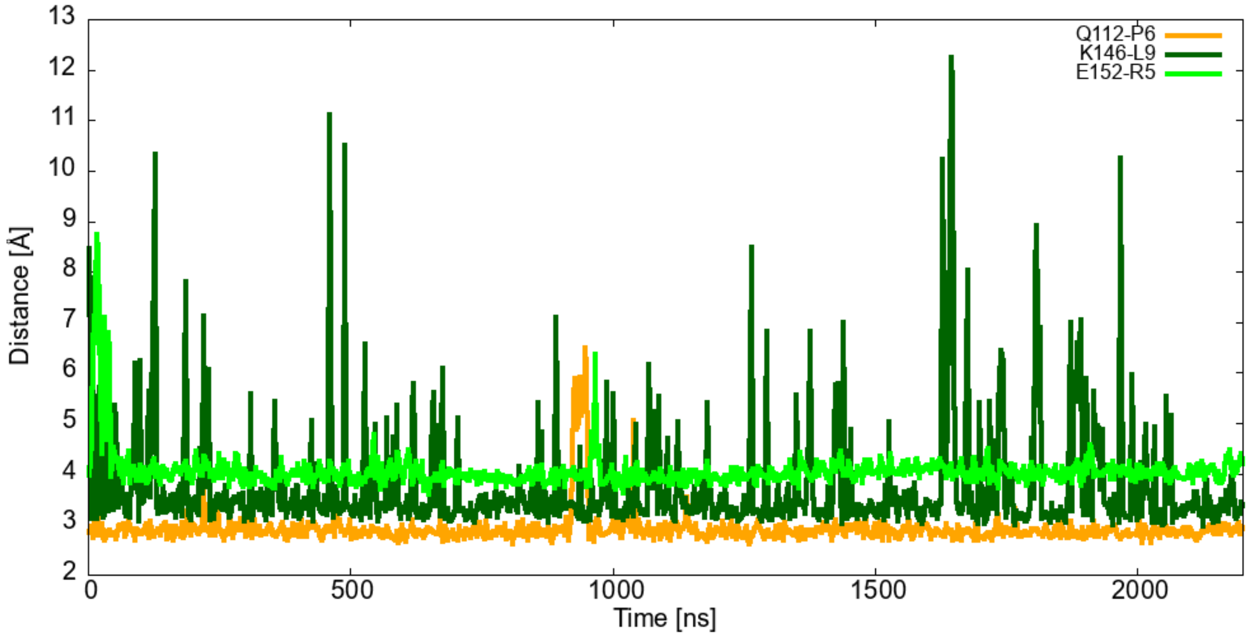

Figure S21. Hydrogen bonds of CD94 protein and HLA-E ligand with the nonameric peptide in **COM⁺** as a function of simulation time: NE2@Gln112^{CD94}-O@Thr6 (orange), NZ@Lys146$^{HLA\text{-}E}$-O@Leu9 (dark-green) and CD@Glu152$^{HLA\text{-}E}$-CZ@Arg5 (lime).

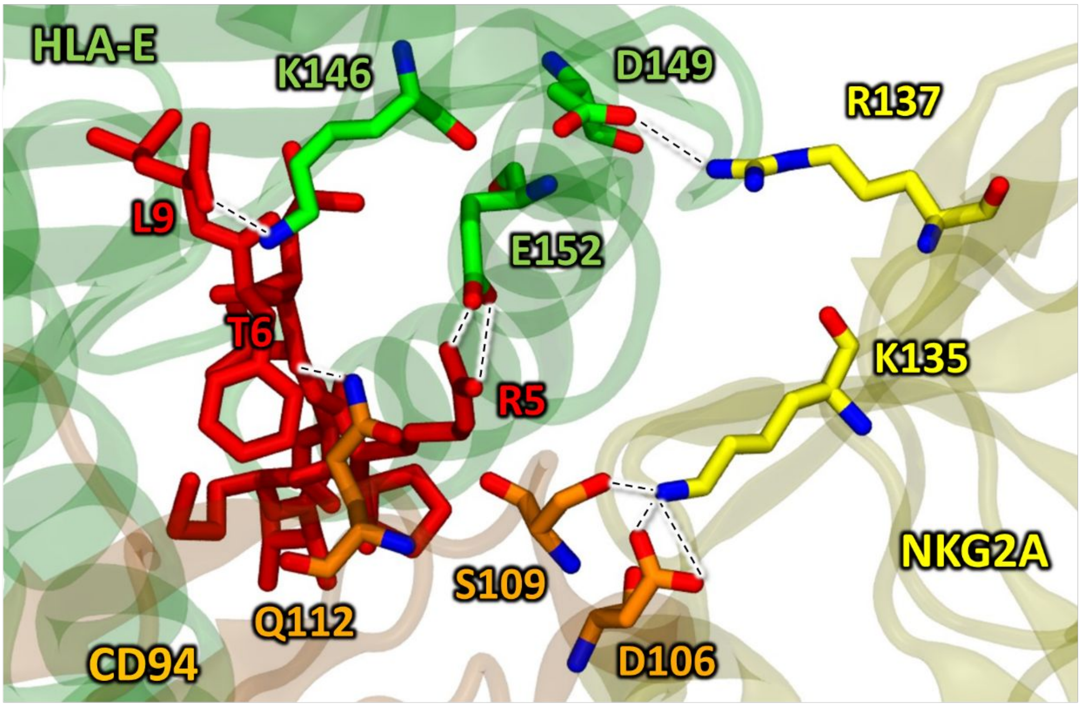

Figure S22. Predominant hydrogen bonds between NKG2A (yellow), CD94 (orange), HLA-E (green) and the nonameric peptide (red) in the **COM+** model.

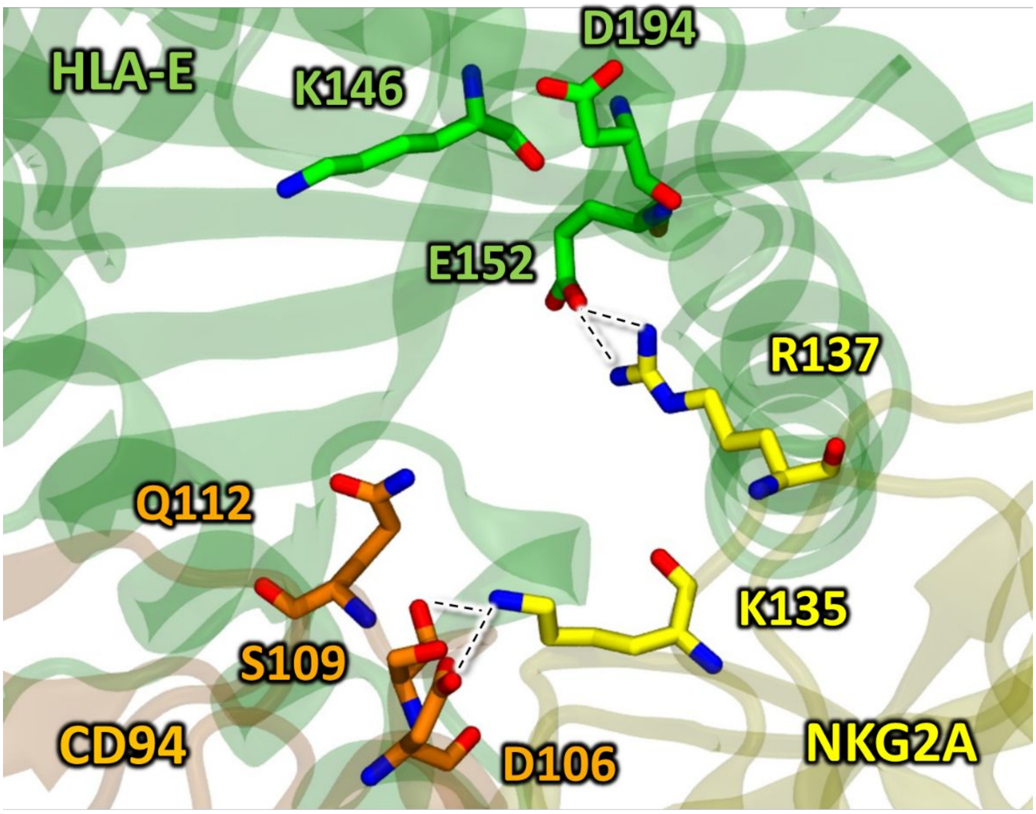

Figure S23. Predominant hydrogen bonds between NKG2A (yellow), CD94 (orange), HLA-E (green) and the nonameric peptide (red) in the **COM-** model.

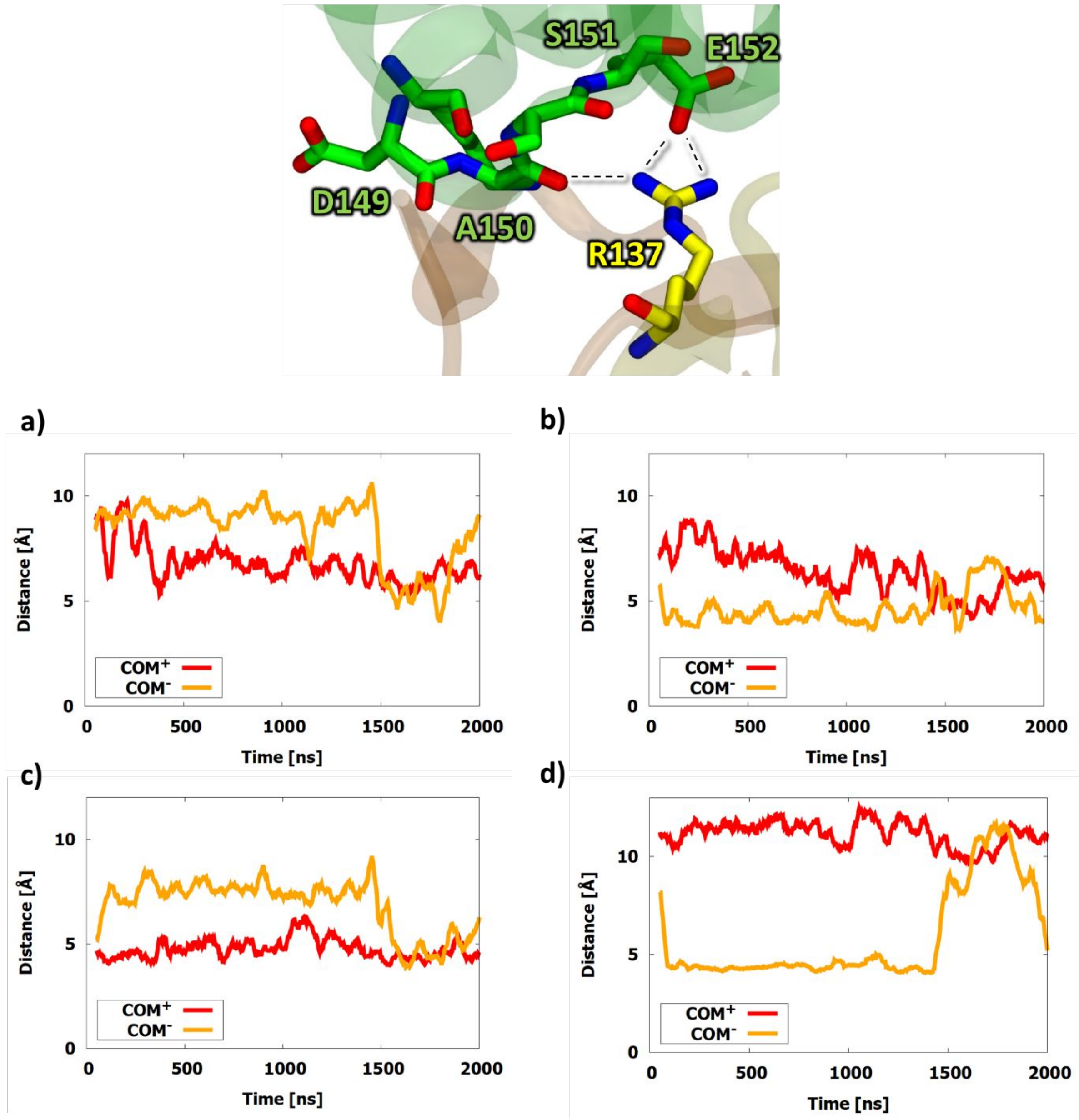

Figure S24. Distance vs time between Arg137^{NKG2A} residue and key HLA-E residues: a) Asp149$^{HLA\text{-}E}$, b) Ala150$^{HLA\text{-}E}$, c) Ser151$^{HLA\text{-}E}$ and d) Glu152$^{HLA\text{-}E}$. Shown above is a snapshot of **COM**$^{-}$, where Arg137^{NKG2A} is seen interacting with Glu152$^{HLA\text{-}E}$ and Ala150$^{HLA\text{-}E}$ more frequently.

Supporting tables

Table S1. Summary of the average distance and persistence of specific hydrogen bonds in **COM$^+$**, **COM$^-$** and **REC** models, highlighted in the paper. Persistence indicates the percentage of frames in which contact is established between residues within a threshold of 4Å.

	**COM$^+$**		**COM$^-$**		**REC**	
	Persistence [%]	Distance [Å]	Persistence [%]	Distance [Å]	Persistence [%]	Distance [Å]
Asp106^{CD94}-Lys135^{NKG2A}	38.5	5.13	49.5	5.05	15.0	7.08
Ser109^{CD94}-Lys135^{NKG2A}	57.7	5.14	54.4	4.10	14.2	8.11
Arg137^{NKG2A}-Asp149^{HLA-E}	7.0	6.56	3.8	8.29		
Arg137^{NKG2A}-Ala150^{HLA-E}	10.4	6.18	45.0	4.70		
Arg137^{NKG2A}-Ser151^{HLA-E}	23.6	4.79	9.3	6.82		
Arg137^{NKG2A}-Glu152^{HLA-E}	0	11.15	39.2	5.57		
Gln112^{CD94}-Thr6^{PEPTIDE}	98.4	2.91				
Lys146^{HLA-E}-Leu9^{PEPTIDE}	79.1	3.64				
Glu152^{HLA-E}-Arg5^{PEPTIDE}	97.6	3.06				

Table S2. MMGBSA per-residue energy decomposition for the nonameric peptide in complex with NKG2A/CD94/HLA-E in the **COM⁺** models.

	$E_{PEPTIDE}$ (kcal/mol)
**Val1**	2.17 ± 2.76
**Met2**	-9.49 ± 1.97
**Ala3**	-1.11 ± 1.08
**Pro4**	-1.76 ± 0.11
**Arg5**	-4.98 ± 3.29
**Thr6**	-2.73 ± 0.40
**Leu7**	-5.97 ± 0.19
**Phe8**	-8.71 ± 0.63
**Leu9**	-8.57 ± 0.33

Table S3. MMGBSA energies of all three models, calculated between NKG2A and CD94 for linker, transmembrane and intracellular regions.

	E_{LINKER} (kcal/mol)	E_{TM} (kcal/mol)	E_{IC} (kcal/mol)
**COM+**	-23.3 ± 4.6	-39.2 ± 5.2	-11.0 ± 4.8
**COM-**	-28.4 ± 5.0	-40.6 ± 4.1	-12.2 ± 4.9
**REC**	-20.6 ± 5.3	-29.0 ± 3.8	-2.3 ± 0.5

Supporting movies

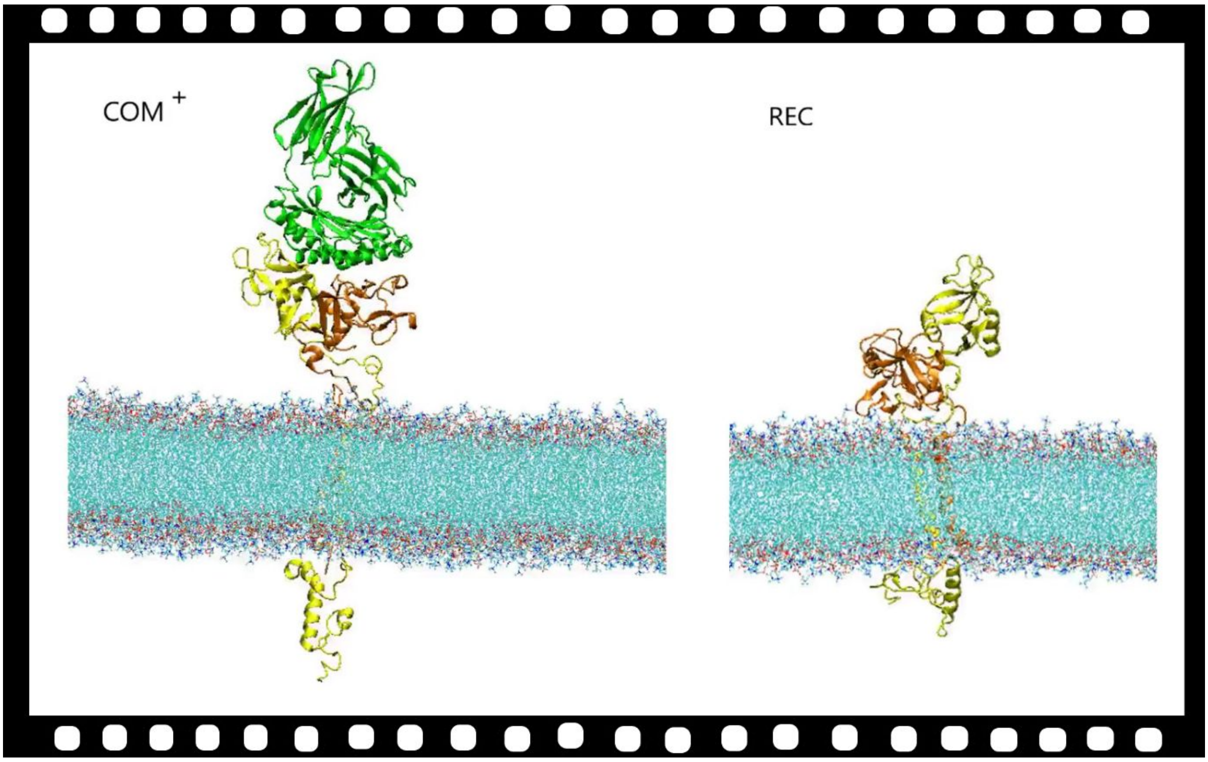

Movie S1. Trajectories of **COM⁺** (left) and **REC** (right) during the 2 µs-long simulation.
